# Foxtail Millet NF-Y Families: Genome-Wide Survey and Evolution Analyses Identified Two Functional Genes Important in Abiotic Stresses

**DOI:** 10.3389/fpls.2015.01142

**Published:** 2015-12-22

**Authors:** Zhi-Juan Feng, Guan-Hua He, Wei-Jun Zheng, Pan-Pan Lu, Ming Chen, Ya-Ming Gong, You-Zhi Ma, Zhao-Shi Xu

**Affiliations:** ^1^Institute of Crop Science, Chinese Academy of Agricultural Sciences (CAAS)/National Key Facility for Crop Gene Resources and Genetic Improvement, Key Laboratory of Biology and Genetic Improvement of Triticeae Crops, Ministry of AgricultureBeijing, China; ^2^Institute of Vegetables, Zhejiang Academy of AgricultureHangzhou, Zhejiang, China; ^3^College of Agronomy, Northwest A&F UniversityYangling, Shaanxi, China

**Keywords:** NF-Y transcription factor, evolution analysis, induced mechanism, gene regulation, physiological trait, drought tolerance, *Setaria italica*

## Abstract

It was reported that Nuclear Factor Y (NF-Y) genes were involved in abiotic stress in plants. Foxtail millet (*Setaria italica*), an elite stress tolerant crop, provided an impetus for the investigation of the NF-Y families in abiotic responses. In the present study, a total of 39 NF-Y genes were identified in foxtail millet. Synteny analyses suggested that foxtail millet NF-Y genes had experienced rapid expansion and strong purifying selection during the process of plant evolution. *De novo* transcriptome assembly of foxtail millet revealed 11 drought up-regulated NF-Y genes. *SiNF-YA1* and *SiNF-YB8* were highly activated in leaves and/or roots by drought and salt stresses. Abscisic acid (ABA) and H_2_O_2_ played positive roles in the induction of *SiNF-YA1* and *SiNF-YB8* under stress treatments. Transient luciferase (LUC) expression assays revealed that SiNF-YA1 and SiNF-YB8 could activate the *LUC* gene driven by the tobacco (*Nicotiana tobacam*) *NtERD10, NtLEA5, NtCAT, NtSOD*, or *NtPOD* promoter under normal or stress conditions. Overexpression of *SiNF-YA1* enhanced drought and salt tolerance by activating stress-related genes *NtERD10* and *NtCAT1* and by maintaining relatively stable relative water content (RWC) and contents of chlorophyll, superoxide dismutase (SOD), peroxidase (POD), catalase (CAT) and malondialdehyde (MDA) in transgenic lines under stresses. *SiNF-YB8* regulated expression of *NtSOD, NtPOD, NtLEA5*, and *NtERD10* and conferred relatively high RWC and chlorophyll contents and low MDA content, resulting in drought and osmotic tolerance in transgenic lines under stresses. Therefore, SiNF-YA1 and SiNF-YB8 could activate stress-related genes and improve physiological traits, resulting in tolerance to abiotic stresses in plants. All these results will facilitate functional characterization of foxtail millet NF-Ys in future studies.

## Introduction

Nuclear Factor Y (NF-Y), also called heme-activated protein (HAP) or CCAAT binding factor (CBF), is a heterotrimeric transcription factor comprised of three distinct subunits: NF-YA (HAP2 or CBF-B), NF-YB (HAP3 or CBF-A), and NF-YC (HAP5 or CBF-C) (Romier et al., 2003). Each subunit is required for DNA binding, subunit association and transcriptional regulation in animals (Sinha et al., 1995). NF-YB and NF-YC initially form a dimer in the cytoplasm and then translocate to the nucleus where they interact with NF-YA and bind CCAAT sites, one of the most common elements in eukaryotic promoters (FitzGerald et al., 2004; Testa et al., 2005). In animals and yeast, each NF-Y subunit is encoded by a single gene and is required for control of growth and development (Mantovani, 1999). Many housekeeping, tissue/development and cell cycle-specific genes are targets of NF-Ys. Not surprisingly, various NF-Y mutations in mouse and *Drosophila* are lethal (Frontini et al., 2004; Yoshioka et al., 2007).

In plants, each of three NF-Y subunits is encoded by multiple genes (Edwards et al., 1998; Keddie et al., 2000). Amplification of the NF-Y family raises the possibility that NF-Ys have evolved new and divergent functions in plants. Several studies showed that NF-Y transcription factors might act as switches in the complex regulatory networks controlling abiotic stress processes. Overexpression of soybean *GmNF-YA3* enhanced drought tolerance and increased sensitivity to exogenous abscisic acid (ABA) by activating ABA-responsive genes in *Arabidopsis* (Ni et al., 2013). Poplar *PdNF-YB7* conferred drought tolerance in *Arabidopsis* and overcame sensitivity to drought stress conferred by the *atnf-yb3* mutant (Han et al., 2013). Overexpression of maize *ZmNF-YB2* increased crop productivity under drought field tests based on the responses of a number of stress-related parameters, including chlorophyll content, stomatal conductance, leaf temperature, reduced wilting, and maintenance of photosynthesis (Nelson et al., 2007). In addition, microarray analysis showed that *Arabidopsis AtNF-YA5* improved drought tolerance by regulating a number of drought- and oxidative-inducible genes (Li et al., 2008). However, the relation of oxidative stress with NF-Y genes was unclear. Therefore, there was a need to investigate whether oxidative stress was involved in the transcriptional regulation of NF-Y genes.

Foxtail millet (*Setaria italica*), an elite stress-tolerant crop, is an important food and fodder grain crop in arid and semi-arid regions of Asia and Africa. However, a collective understanding of NF-Y families in foxtail millet has not been established under abiotic stress conditions. In the present study, we characterized three unique “Si” NF-Y families in foxtail millet, and found that *SiNF-YA1* and *SiNF-YB8* could enhance stress tolerance in tobacco. This research might serve as an entree to obtain rapid progress in determining the roles of foxtail millet NF-Y genes in abiotic stress responses.

## Materials and methods

### Discovery and annotation of NF-Y families

Database BLASTP searches were performed to identify foxtail millet NF-Y members using the known NF-Y conserved core regions of *Arabidopsis* and rice. The known NF-Y sequences were retrieved from the *Arabidopsis* (http://www.arabidopsis.org) and rice (http://rice.plantbiology.msu.edu/) databases. The Hidden Markov Model (HMM) profiles of known NF-Y sequences were also performed to identify the potential foxtail millet NF-Ys (*E*-value = 0.01). The whole NF-Y protein sequences of foxtail millet were downloaded from the foxtail millet database (http://www.phytozome.org/) (Release 9.0) and plant transcription factor database (http://planttfdb.cbi.pku.edu.cn/). Further, all the sequences identified from BLASTP search and HMM search were queried against Pfam (version 20.0, http://www.sanger.ac.uk/Software/Pfam/) and ProDom (http://prodom.prabi.fr/prodom/current/html/home.php) to confirm their identity as potential NF-Y subunits. The redundant sequences or sequences that do not contain the known core regions were removed manually.

### Homology modeling of NF-Y proteins

NF-Y proteins were searched against the Protein Data Bank (PDB) (http://www.rcsb.org/pdb/) by BLASTP (with the default parameters) to identify the best template having similar sequence and known three-dimensional structure. The data were fed into Phyre2 (Protein Homology/AnalogY Recognition Engine; http://www.sbg.bio.ic.ac.uk/phyre2) for predicting the protein structure by homology modeling under the “intensive” mode. The protein structures of NF-Y proteins were modeled at 90% confidence.

### Multiple alignments, phylogenetic tree and gene structure

Multiple sequence alignments were performed using ClustalW with gap open and gap extension penalties of 10 and 0.1, respectively (Thompson et al., 1997). Protein sequence motifs were identified using multiple EM for motif elicitation (MEME) (http://meme.nbcr.net/meme3/meme.html). Discovered MEME motifs (≤1E-30) were searched in the InterPro database with InterProScan (Quevillon et al., 2005). NF-Y subunit-conserved core regions and other identified motifs were used to create consensus logos using WebLogo (http:// weblogo.berkeley.edu/) and default program parameters.

Phylogenetic analysis was undertaken based on the bootstrap neighbor-joining (NJ) method by MEGA4 with the following parameters: Kimura two-parameter model, pairwise gap deletion and 1000 bootstraps (Tamura et al., 2007). Regulatory elements were analyzed using PLACE (http://www.dna.affrc.go.jp).

### Chromosomal distribution, genome synteny, and gene duplication

Specific chromosomal positions of the NF-Y genes were plotted according to ascending order of physical position from the short arm telomere to the long arm telomere and finally displayed using MapInspect (He et al., 2012). Segmental duplications were calculated based on the method of Plant Genome Duplication Database (http://chibba.pgml.uga.edu/duplication/). Tandem duplications were identified manually. Adjacent genes of same sub-group tightly linked within 20 kb of each other and the identity of the genes ≥80% are considered as tandem duplicated genes. The syntenic relationships between foxtail millet and other plant species (*Arabidopsis*, rice and *Brachypodium*) were then drawn using Circos v0.55 (http://circos.ca/). BLASTP was also performed to ensure unique relationship between the orthologous gene pairs and all hits with *E*-value ≤ 1E-5 and at least 80% homology were considered significant. The Ka/Ks ratios were estimated for ortholog NF-Y gene pairs through CODEML program in PAML interface tool of PAL2NAL (http://www.bork.embl.de/pal2nal/). Ka and Ks were numbers of non-synonymous and synonymous substitutions per site, respectively. Ka/Ks > 1 indicated gene evolution under positive selection, Ka/Ks < 1 indicated purifying (stabilizing) selection and Ka/Ks = 1 suggested a lack of selection or possibly a combination of positive and purifying selection at different points within the gene that canceled each other out.

### *De novo* transcriptome assembly

Seeds of foxtail millet cultivar Yugu 1 were used for all experiments. For drought treatment, 21-day-old foxtail millet seedlings were not watered for 1 week in soil (28°C day/20°C night, 16 h photoperiod, 65% relative humidity). The seedlings with the same growth state were used as the control. Construction of subtracted cDNA libraries, sequencing, data analysis of expressed sequence tags (ESTs), differential screening of ESTs by microarray analysis and statistical analysis were performed as described by Puranik (Puranik et al., 2011). The normalized data was subjected to fold difference calculation. ESTs that showed *E*-value ≤ 10^−5^ and more than 100 nucleotides in length and fold difference ≥ 2 were considered significant. All values were mean of three independent experiments. For each sample, a total of 40.5 million reads were obtained.

### Stress and inhibitor treatments for quantitative RT-PCR (qRT-PCR)

For stress treatments, 21-day-old seedlings were exposed to 10% PEG 6000 (drought stress), 150 mM NaCl (salt stress), 200 mM mannitol (osmotic stress), and 30 mM H_2_O_2_ (oxidative stress) for 3 h. Roots, stems and leaves were immediately frozen in liquid nitrogen and stored at −80°C after each treatment. For inhibitor or scavenger treatment, ABA inhibitor fluridone (BioDee, China) or H_2_O_2_ scavenger dimethyl thiourea (DMTU) (BioDee, China) was used. Foxtail millet seedlings were pretreated with 100 μM fluridone or 10 mM DMTU for 6 h to stop the production of ABA or H_2_O_2_, followed by exposure to dehydration treatment for 2, 6, or 12 h. RNA was extracted using Trizol reagent (TaKaRa, Japan) and the first strand cDNA was synthesized with a PrimeScript 1st Strand cDNA Synthesis kit (TaKaRa, Japan). SYBR Green (RealMasterMix, Tiangen, China) and ABI 7300 (Applied Biosystem, USA) were used to monitor the kinetics of PCR product formation in qRT-PCR. The amounts of transcript accumulated for SiNF-Y genes normalized to the internal control *Actin* (AF288226.1) were determined using the 2^−ΔΔCT^ method. For obtaining reproducible results, each experiment was repeated three times.

### Subcellular localization assay

An expression vector p16318GFP with a green fluorescent protein (GFP) tag was constructed for subcellular localization analysis. The SiNF-Y open reading frame (ORF), lacking a stop codon, was amplified and fused to the N-terminal end of GFP under control of the CaMV 35S promoter. The reconstruction vector was bombarded into onion epidermal cells by a particle gun (Xu et al., 2007; Liu et al., 2013). GFP signal in epidermal cells was visualized by a confocal laser scanning microscope with a Fluar 10X/0.50 M27 objective lens and SP640 filter (Leica Microsystem, Heidelberg, Germany).

### Generation of tobacco lines

The target gene (*SiNF-YA1* or *SiNF-YB8*) was cloned into the pBI121 vector driven by the CaMV 35S promoter, and transformed into tobacco (W38 genetic background) using the *Agrobacterium*-mediated transformation method (Xu et al., 2009). Seeds from transformed tobacco plants were plated in 50 mg/L kanamycin (Kan) selection medium in a growth chamber (16 h light/8 h darkness, 70% relative humidity, 20°C). Homozygous T3 transformed tobacco were selected from a T2 population segregating and confirmed by qRT-PCR for further abiotic stress tolerance analysis. The β*-tubulin* gene was used as an internal control for qRT-PCR assay.

### Abiotic stress treatments of transgenic tobacco lines

Three independent transgenic tobacco lines with higher expression of target gene were used to perform abiotic stress tolerance assay. For seed germination assays, 50 sterile seeds were cultured on MS agar plates supplemented with PEG, NaCl and mannitol. Germination rates were scored at radicle emergence. To examine root morphologies, 1-week-old tobacco seedlings were transferred to MS agar plates supplemented with PEG, NaCl and mannitol and cultured vertically for 6 d under the above regime. Root length, fresh weight, and dry weight were measured. For drought treatment in soil, 1-week-old seedlings were not watered for 1 week, and then re-watered for 2 weeks under the above regime. For salt treatment in soil, 2-week-old seedlings were watered with 200 mM NaCl for 1 week and then re-watered normally for 2 weeks. Before re-watering, leaves were harvested for RNA extraction and measurement of relative water content (RWC), chlorophyll content, superoxide dismutase (SOD), peroxidase (POD), catalase (CAT), and malondialdehyde (MDA) activity (Hu et al., 2013; Zhang et al., 2013). The constitutive β*-tubulin* transcript, as an internal control, was used to quantify the relative expression levels of stress responsive marker genes using a qRT-PCR assay. Three independent experiments were accomplished and for each sample three technical replicates were analyzed.

### Transient luciferase (LUC) assay

Transcription activity of SiNF-YA1 or SiNF-YB8 against promoters of stress responsive marker genes was performed using dual luciferase assay of transiently transformed tobacco leaves (Huang et al., 2013). Marker gene promoters from tobacco were amplified by thermal asymmetric inter-laced PCR (Tail-PCR) and subcloned into the transient expression reporter vector pGreenII0800-LUC which contained the CaMV 35S promoter-REN cassette and the promoterless-LUC cassette. pBI121-SiNF-Y vector was used as effector construct as previously described (Yotsui et al., 2013). No-effector construct was used as control. Firefly luciferase and renilla luciferase were assayed using the dual luciferase assay reagents (Promega, USA). Data was collected as the ratio of LUC/REN. Three independent experiments were accomplished and for each sample three technical replicates were analyzed.

### Statistical analysis

Statistical analyses were performed using the software in Excel. Analysis of variance was used to compare the statistical difference based on Student's *t*-test, at a significant level of 0.01 < *P* < 0.05, *P* < 0.01.

### Primers

All primers used for vector construction, PCR, RT-PCR and qRT-PCR assays for all target genes are listed in Supplementary Table S1.

## Results

### Identification and multiple alignments of NF-Y families in foxtail millet

Previously, *Arabidopsis* NF-Y families, including 10 NF-YA, 13 NF-YB, and 13 NF-YC genes were characterized. Full-length proteins and conserved regions of all 36 *Arabidopsis* NF-Ys were used to BLAST the foxtail millet database and identified 10 NF-YA, 15 NF-YB, and 14 NF-YC genes in the foxtail millet genome (Table 1; Supplementary Datasets S1, S2).

**Table 1:** **Annotation of foxtail millet NF-Y families**.

**NF-YA family**		**NF-YB family**		**NF-YC family**	
**Name**	**IBI**	**Name**	**IBI**	**Name**	**IBI**
*SiNF-YA1*	Si037045 m	*SiNF-YB1*	Si014159 m	*SiNF-YC1*	Si018151 m
*SiNF-YA2*	Si037269 m	*SiNF-YB2*	Si003159m	*SiNF-YC2*	Si007127 m
*SiNF-YA3*	Si023231 m	*SiNF-YB3*	Si023400 m	*SiNF-YC3*	Si020107 m
*SiNF-YA4*	Si019645 m	*SiNF-YB4*	Si038696 m	*SiNF-YC4*	Si032636 m
*SiNF-YA5*	Si036728 m	*SiNF-YB5*	Si031069 m	*SiNF-YC5*	Si037200 m
*SiNF-YA6*	Si030663 m	*SiNF-YB6*	Si020091 m	*SiNF-YC6*	Si015032 m
*SiNF-YA7*	Si022607 m	*SiNF-YB7*	Si014286 m	*SiNF-YC7*	Si015775 m
*SiNF-YA8*	Si032469m	*SiNF-YB8*	Si018339 m	*SiNF-YC8*	Si004603m
*SiNF-YA9*	Si036465m	*SiNF-YB9*	Si008357 m	*SiNF-YC9*	Si022720 m
*SiNF-YA10*	Si024641 m	*SiNF-YB10*	Si004874 m	*SiNF-YC10*	Si026768 m
		*SiNF-YB11*	Si024597 m	*SiNF-YC11*	Si026839 m
		*SiNF-YB12*	Si004211 m	*SiNF-YC12*	Si022020 m
		*SiNF-YB13*	Si007336 m	*SiNF-YC13*	Si012136 m
		*SiNF-YB14*	Si007865 m	*SiNF-YC14*	Si023636 m
		*SiNF-YB15*	Si031288 m		

**Figure 1:**
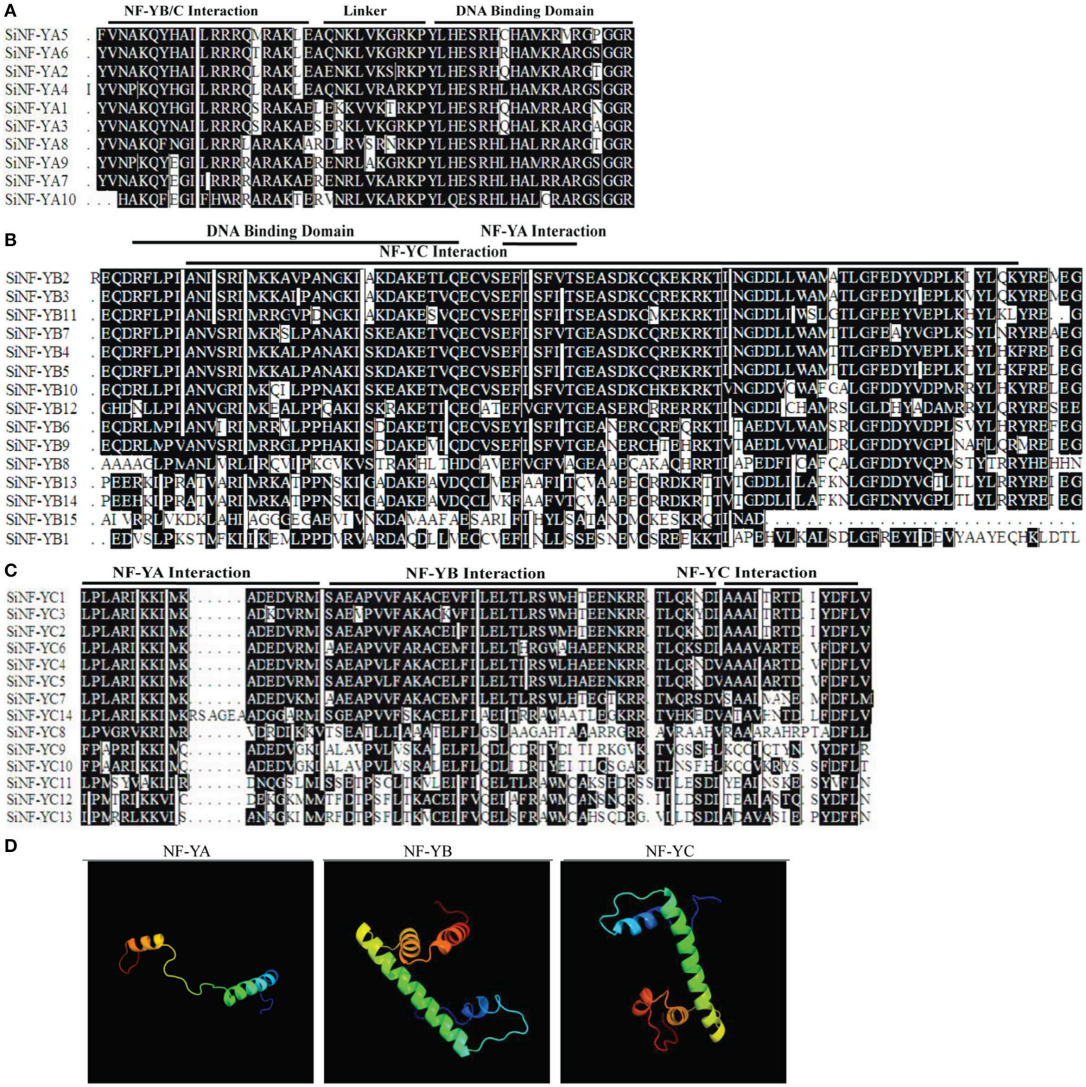
**Multiple alignments of foxtail millet NF-Y family members and predicated structures of NF-Y conserved regions**. **(A)** Multiple alignments of the SiNF-YA family. **(B)** Multiple alignments of SiNF-YB family. **(C)** Multiple alignments of SiNF-YC family. Amino acids in black boxes/white letters are identical in at least 50% of all aligned sequences. **(D)** Predicated structures of NF-Y conserved regions.

Multiple alignments showed that foxtail millet NF-Y proteins had the conserved regions and relatively variable N-terminal or C-terminal transcriptional regulation domains (Figures 1A–C; Supplementary Figures S1, S2). Fasta files for full length SiNF-Y proteins and conserved regions were provided in Supplementary Datasets S1, S2, respectively. The conserved core region of SiNF-YA subunits was comprised of two sub-domains, one domain for NF-YB/C interaction and one domain for DNA contact (Figure 1A). Cross-kingdom conservation was identified in *Arabidopsis*, rice, wheat and *Brachypodium* NF-YA members (Supplementary Figure S1A). Like the histone fold motif (HFM) of the core histone H_2_B, the core regions of SiNF-YB subunits contained domains for DNA binding and protein-protein interactions (Maity and de Crombrugghe, 1992; Dorn et al., 1997; Figure 1B). SiNF-YC subunits were also characterized by a core histone, but the core histone was more similar to H_2_A than H_2_B (Dorn et al., 1997; Figure 1C). There was conservation of this theme throughout the plant lineage (Supplementary Figures S1B,C).

The predicted secondary structure of NF-Y proteins was predominantly comprised of α-helices and coils (Figure 1D). The SiNF-YA1 structure was modeled at the conserved core region comprising of two functionally distinct domains, the N-terminal domain for subunit interaction with the NF-YB/NF-YC heterodimer and the C-terminal domain for DNA binding site recognition. The conserved core region of the modeled SiNF-YB8 protein was comprised of regions required for DNA binding or subunit interactions for NF-YA and NF-YC subunits. The conserved core region of the modeled SiNF-YC12 protein was comprised of regions required for DNA binding or subunit interactions for NF-YA, as well as NF-YB subunit. The predicted protein structures were considered highly reliable and offered a preliminary basis for understanding the molecular functions of NF-Y proteins.

Eight conserved motifs outside the conserved core region were identified in the NF-Ys of foxtail millet, *Arabidopsis*, rice, wheat, and *Brachypodium* (Supplementary Figures S1, S2; Supplementary Table S2). In NF-YA subunits, we found one conserved motif 8 (FFTPLP) at the C-terminus in monocots (Supplementary Figures S1A, S2A; Supplementary Table S2A) except reported motif 3, motif 5 (RVPLP), motif 6 (DPYYG) and motif 9 (HPQ) (Stephenson et al., 2007; Thirumurugan et al., 2008; Siefers et al., 2009). In NF-YB subunits, SiNF-YB4, SiNF-YB5, SiNF-YB13, and SiNF-YB14 contained motif 5 (MPDSDNDSG) and motif 6 (MMMMGQPMYGSP) which were conserved only in monocots (Supplementary Figures S1B, S2B; Supplementary Table S2B). In NF-YC subunits, SiNF-YC4 and SiNF-YC5 contained motif 7 (FPAARIKKIM) at the N terminus, whereas AtNF-YC1, AtNF-YC2, AtNF-YC3, AtNF-YC4, and AtNF-YC9 contained this motif at the C terminus (Supplementary Figures S1C, S2C; Supplementary Table S1C). The existence of these motifs only in monocots might imply different functions from dicots.

### Phylogenetic and gene structure analysis

**Figure 2:**
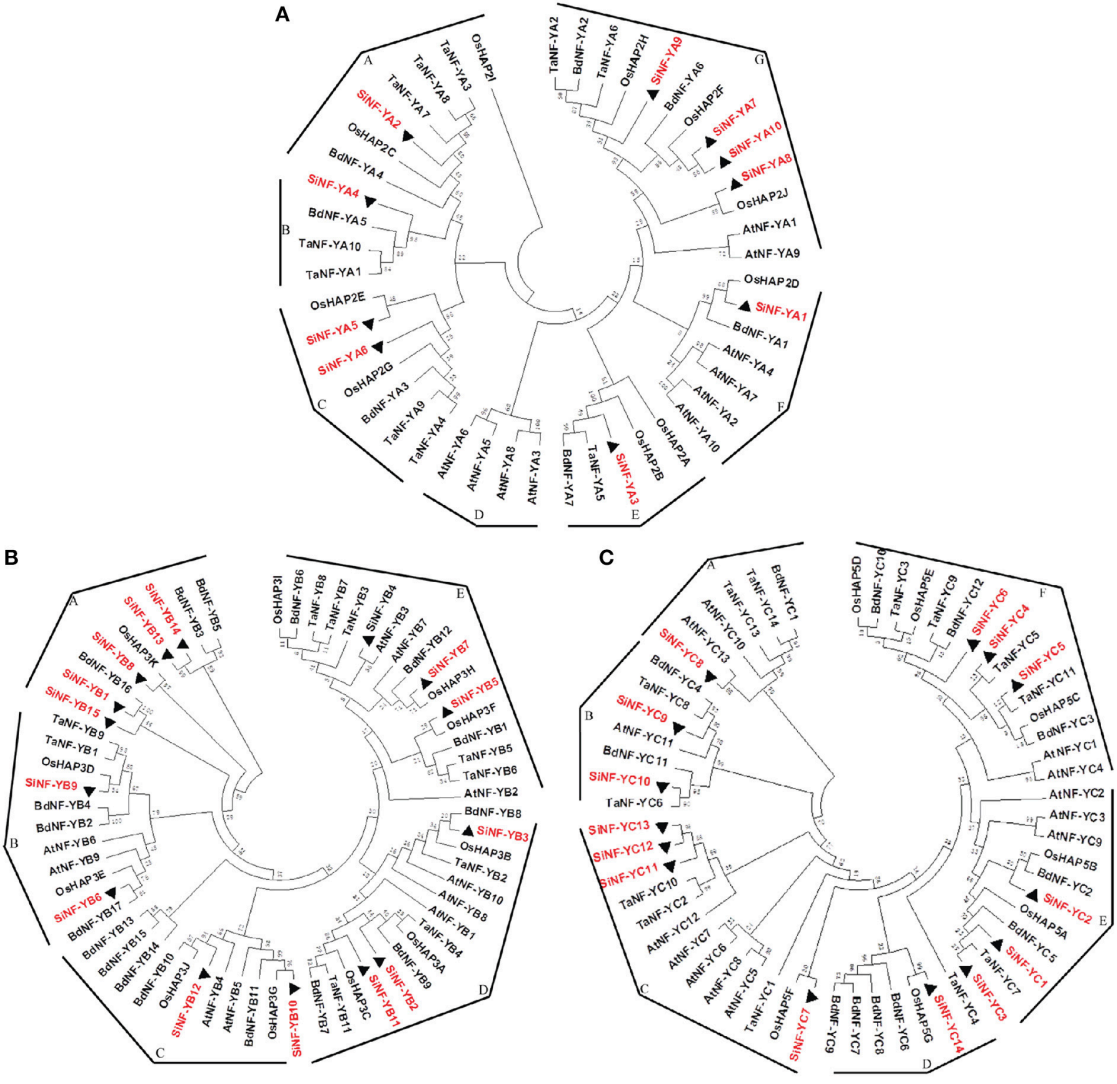
**Phylogenetic trees of NF-Y families in foxtail millet, *Arabidopsis*, rice, wheat, and *Brachypodium*. (A)** NF-YA phylogenetic trees. **(B)** NF-YB phylogenetic trees. **(C)** NF-YC phylogenetic trees. Full length proteins are aligned using ClustalX software and subjected to phylogenetic analysis using the neighbor-joining method method with 1000 resampling replicates. Bootstrap values (%) based on 1000 replicates are indicated beside the nodes. SiNF-Ys are highlighted in red and triangle. The tree is divided into groups designated as A–G.

To derive orthologous relationships of NF-Ys, the evolutionary relationships of foxtail millet NF-Ys were compared with other plant genomes (Figure 2). This enabled the classification of the NF-Y family into 7 groups for NF-YA subunits (Groups A–G), 5 groups for NF-YB subunits (Groups A–E) and 6 groups for NF-YC subunits (Groups A–F). SiNF-YA subunits were generally found on the tree as a series of paralogs that were all roughly equidistant from each other (Figure 2A). This was in contrast to the SiNF-YB and SiNF-YC subunits where there were clear blocks of proteins that were more closely related, or additional blocks that were considerably more divergent (Figures 2B,C).

**Figure 3:**
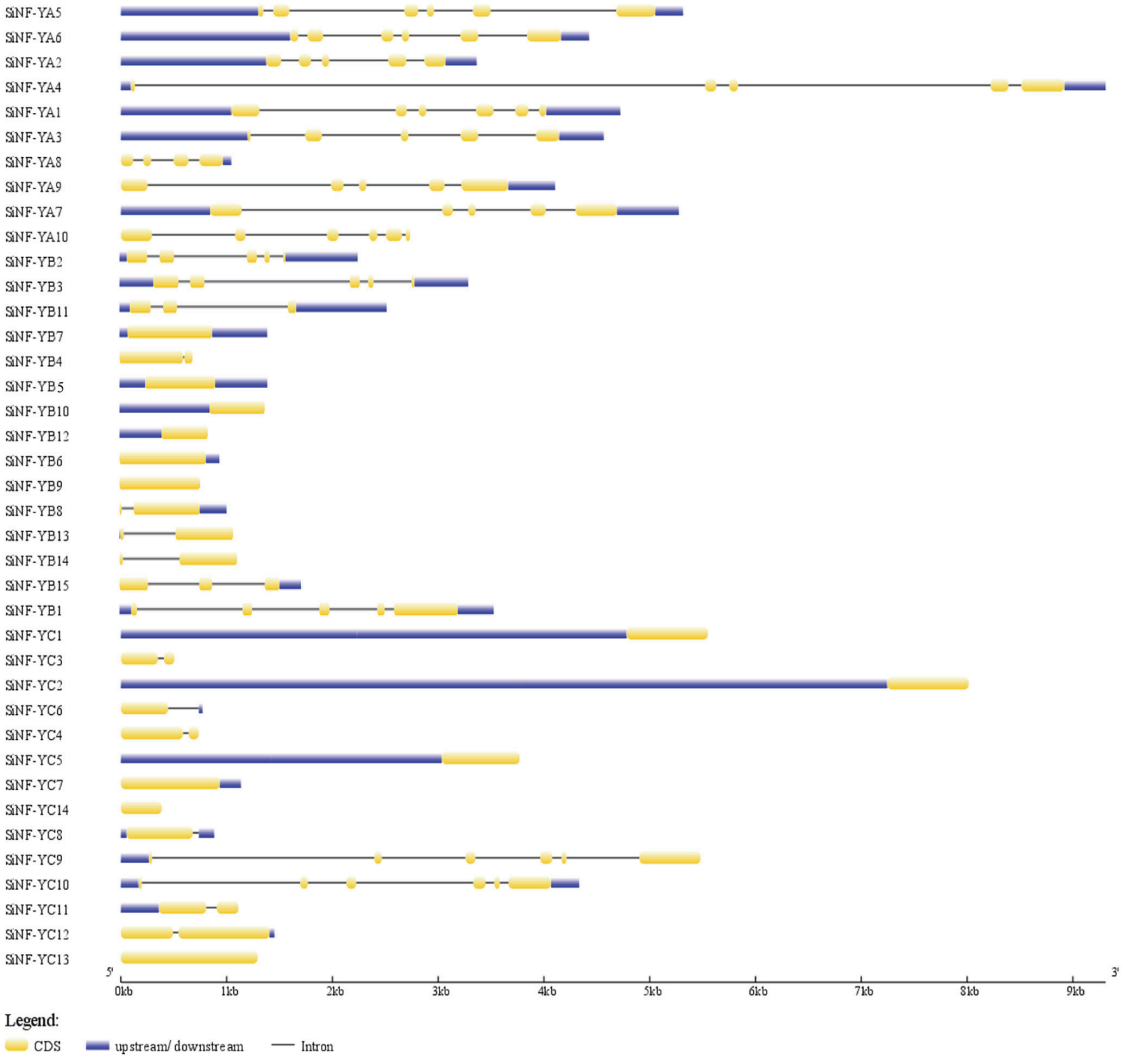
**Gene structures of foxtail millet NF-Y family members**. Exons and introns are represented by yellow boxes and black lines, respectively. The sizes of exons and introns can be estimated using the scale bar at the bottom.

Analysis of gene structure might provide some information about the evolutionary mechanism underlying the genesis of gene families. All NF-YA genes were interrupted by introns. There are 4–5 introns embedded in NF-YA genes, except 3 introns in *SiNF-YA8* (Figure 3). No intron was present in 6 members from the NF-YB family, and other members contained 1–4 introns. There were 6 NF-YC members containing no or only one intron, and others containing 5 introns (Figure 3). All introns imbeded in NF-Ys followed the GT-AG splicing rule, and each of the last exons included a stop codon. Interestingly, several pairs of most homologs were characterized by different gene structures in SiNF-Y subfamilies, such as NF-YA1/A3, NF-YA7/A10, NF-YB4/B5, NF-YB1/B15, NF-YC1/C3, NF-YC4/C5, and NF-YC12/C13 (Figure 3). These results suggested that members of SiNF-Y subfamilies might be active and constantly evolving.

### Chromosomal distribution, genome synteny, and gene duplication analysis

**Figure 4:**
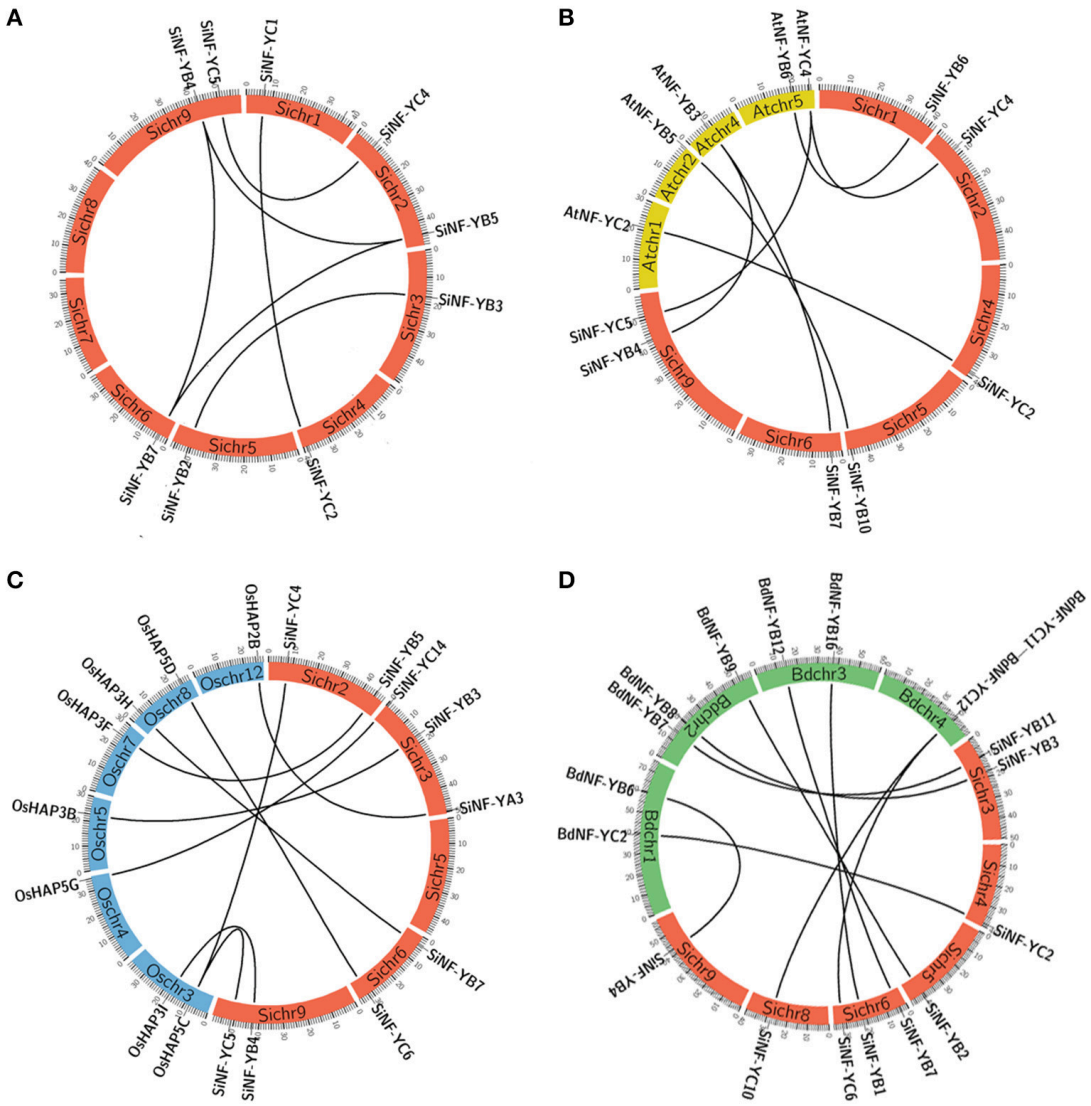
**Synteny analysis of NF-Y genes between foxtail millet, *Arabidopsis*, rice, and *Brachypodium*. (A)** Synteny analysis of NF-Ys in foxtail millet (Si). **(B)** Synteny analysis of NF-Ys between foxtail millet and *Arabidopsis* (At). **(C)** Synteny analysis of NF-Ys between foxtail millet and rice (Os). **(D)** Synteny analysis of NF-Ys between foxtail millet and *Brachypodium* (Bd). The positions of NF-Y genes are depicted in the chromosomes of foxtail millet (red bands of Sichr), *Arabidopsis* (yellow bands of Atchr), rice (blue bands of Oschr) and *Brachypodium* (green bands of Bdchr), respectively. Lines are drawn to connect duplicated or orthologous gene pairs.

Gene duplication events play crucial roles in the amplification of gene family members in the genome. *In silico* mapping revealed an uneven distribution of the NF-Y genes on all 9 chromosomes of foxtail millet (Supplementary Figure S3). Chromosome 3 contained the highest number of SiNF-Y genes, while the lowest numbers were distributed on chromosomes 7 and 8. SiNF-YA genes were mainly located on chromosomes 2, 3, and 9. SiNF-YB and SiNF-YC genes were randomly distributed on all chromosomes. To further understand the expansion mechanism of the SiNF-Y genes, the gene duplication events were analyzed. As shown in Figure 4A and Supplementary Table S3A, 10 pairs of SiNF-Ys were identified in the same syntenic blocks, including 6 segmental duplication events (*SiNF-YB2/B3, SiNF-YB4/B5, SiNF-YB4/B7, SiNF-YB5/B7, SiNF-YC1/C2*, and *SiNF-YC4/C5*) between different chromosomes and the other 4 duplication events (*SiNF-YA7/A10, SiNF-YB6/B8, SiNF-YB9/B13/B14*, and *SiNF-YB10/B12*) within the same chromosome. These results suggested that segmental and tandem duplication events play significant roles in the expansion of SiNF-Y genes.

To further explore the evolutionary process of SiNF-Y genes, genome synteny of the NF-Ys among foxtail millet, *Arabidopsis*, rice and *Brachypodium* were performed. Among them, 7 pairs (*SiNF-YB4/AtNF-YB3, SiNF-YB6/AtNF-YB6, SiNF-YB7/AtNF-YB3, SiNF-YB4/AtNF-YB3, SiNF-YC2/AtNF-YC*2, *NF-YC4/AtNF-YC4*, and *SiNF-YC5/AtNF-YC4*) existed in both foxtail millet and *Arabidopsis* genomes (Figure 4B, Supplementary Table S3B). Ten pairs (*SiNF-YA3/OsHAP2B, SiNF-YB3/OsHAP3B, SiNF-YB4/OsHAP3I, SiNF-YB5/OsHAP3F, SiNF-YB4/OsHAP3I, SiNF-YC1/OsHAP5A, SiNF-YC4/OsHAP5C, SiNF-YC5/OsHAP5C, SiNF-YC6/OsHAP5D*, and *SiNF-YC14/OsHAP5G*) were identified to exhibit synteny with their homologs of rice (Figure 4C; Supplementary Table S3C). Nine pairs (*SiNF-YB1/BdNF-YB16, SiNF-YB2/BdNF-YB9, SiNF-YB3/BdNF-YB8, SiNF-YB4/BdNF-YB6, SiNF-YCB7/BdNF-YB12, SiNF-YB11/BdNF-YB7, SiNF-YC2/BdNF-YC2, SiNF-YC6/BdNF-YC12*, and *SiNF-YC10/BdNF-YC11*) existed in foxtail millet and *Brachypodium* genomes (Figure 4D; Supplementary Table S3D). Certainly, some SiNF-Y genes were not mapped to any syntenic blocks with other plant NF-Ys. This can be explained by the fact that foxtail millet and *Arabidopsis*, rice, or *Brachypodium* chromosomes have undergone extensive rearrangements and fusions that possibly lead to selective gene loss.

The ratios of non-synonymous (Ka) vs. synonymous (Ks) substitution rate (Ka/Ks) for duplicated gene-pairs as well as between orthologous gene-pairs of SiNF-Ys with those of *Arabidopsis*, rice and *Brachypodium* were < 1 (Supplementary Table S3). The average Ka/Ks value was maximum between rice and foxtail millet and least for *Arabidopsis*-foxtail millet gene pairs. Remarkably, the NF-Y gene pairs between *Arabidopsis* and foxtail millet (average Ka/Ks = 0.0046) appear to have undergone strong purifying selection in comparison to foxtail millet-rice (0.1078). These genome synteny data along with Ka/Ks data would assist in understanding the evolution of NF-Y genes in monocot and dicot species.

### Abiotic stress expression profiles of NF-Y families

**Figure 5:**
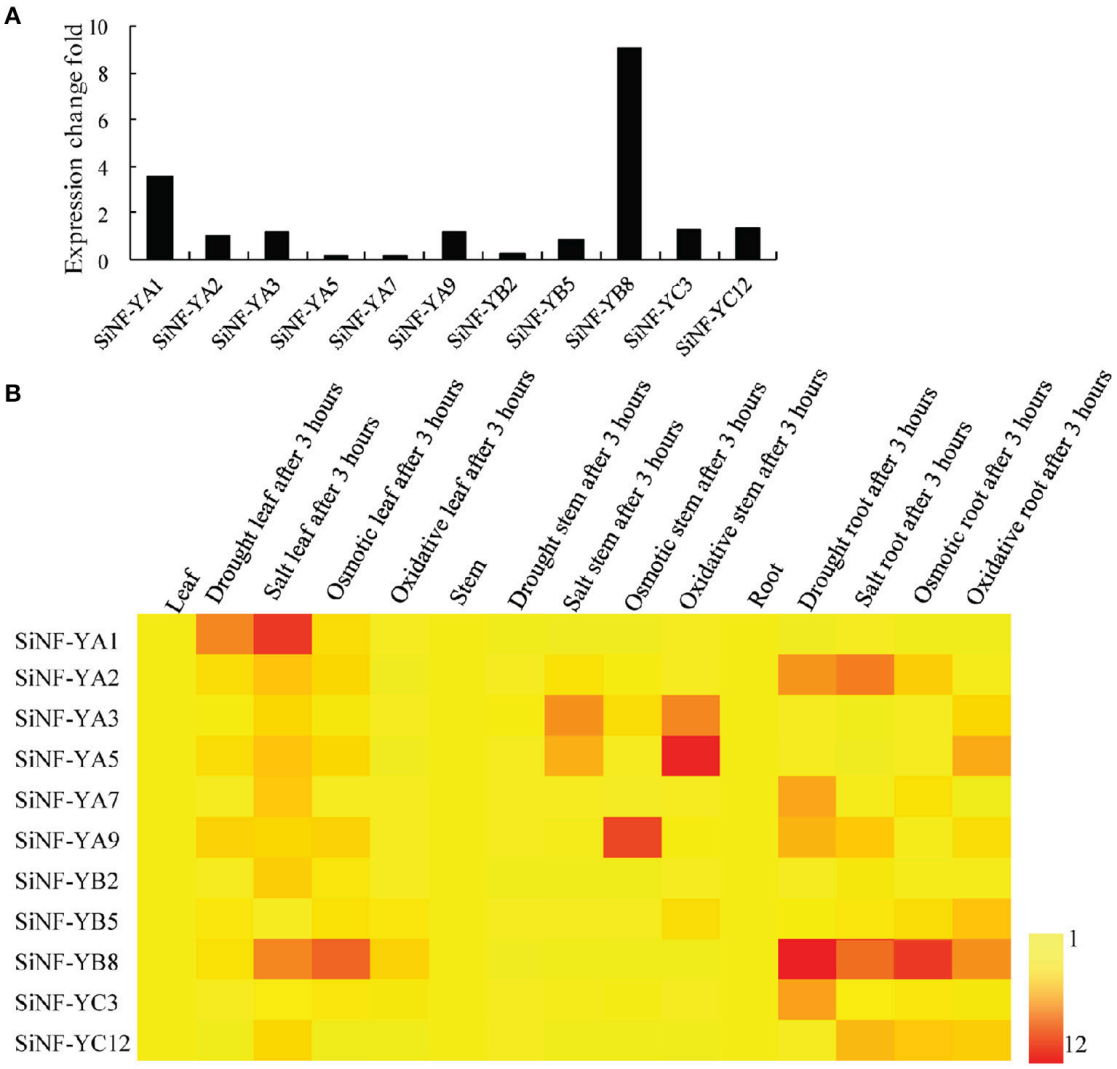
**Expression pratterns of foxtail millet NF-Y genes. (A)** Drought-induced NF-Y genes from the *de novo* transcriptome assembly of foxtail millet under drought treatment. **(B)** Expression profiles of foxtail millet NF-Y genes in different tissues (roots, stems, and lesves) and in response to various stress (drought, salt, osmotic, and oxidative stress) treatments for 3 h.

To investigate drought responsive mechanisms of NF-Y families in foxtail millet, *de novo* transcriptome assembly of foxtail millet with drought treatment was performed. Six NF-YAs (*NF-YA1, NF-YA2, NF-YA3, NF-YA5, NF-YA7*, and *NF-YA9*), three NF-YBs (*NF-YB2, NF-YB5*, and *NF-YB8*) and two NF-YCs (*NF-YC3* and *NF-YC12*) showed up-regulation in expression level (Figure 5A; Supplementary Table S4). Among them, *SiNF-YA1* and *SiNF-YB8* showed the most up-regulation after drought treatment, 3.89 and 9.06 fold, respectively.

Accumulating evidence indicated that NF-Ys were involved in responses to various abiotic stresses. The expression profiles of these 11 SiNF-Ys in responses to four abiotic stresses (drought, salt, mannitol, and oxidative stress) in three tissues (roots, stems, and leaves) were investigated using qRT-PCR. As shown in a heat map (Figure 5B), each NF-Y gene member showed a differential expression pattern. *SiNF-YA1* and *SiNF-YA2* were mainly up-regulated in leaves and roots, respectively, under drought and salt treatments. *SiNF-YA3* and *SiNF-YA5* were mainly induced in stems by salt and oxidative treatments. *SiNF-YA9* was mainly up-regulated in stems under osmotic treatment. Among three SiNF-YB genes, *SiNF-YB2* and *SiNF-YB5* were only weakly up-regulated (no more than 3 fold) under salt and oxidative treatments. By comparison, *SiNF-YB8* showed notable changes, mainly in leaves and roots, under drought and osmotic stress treatments. For the two SiNF-YC genes, *SiNF-YC1* was up-regulated only in roots under drought treatment. *SiNF-YC12* was up-regulated (no more than 4 fold) under salt, mannitol, and H_2_O_2_ treatments. Two NF-Y genes, *SiNF-YA1* and *SiNF-YB8* with notable expression changes, were selected for further study.

### ABA and H_2_O_2_ were involved in induction of *SiNF-YA1* and *SiNF-YB8* under stress treatments

**Figure 6:**
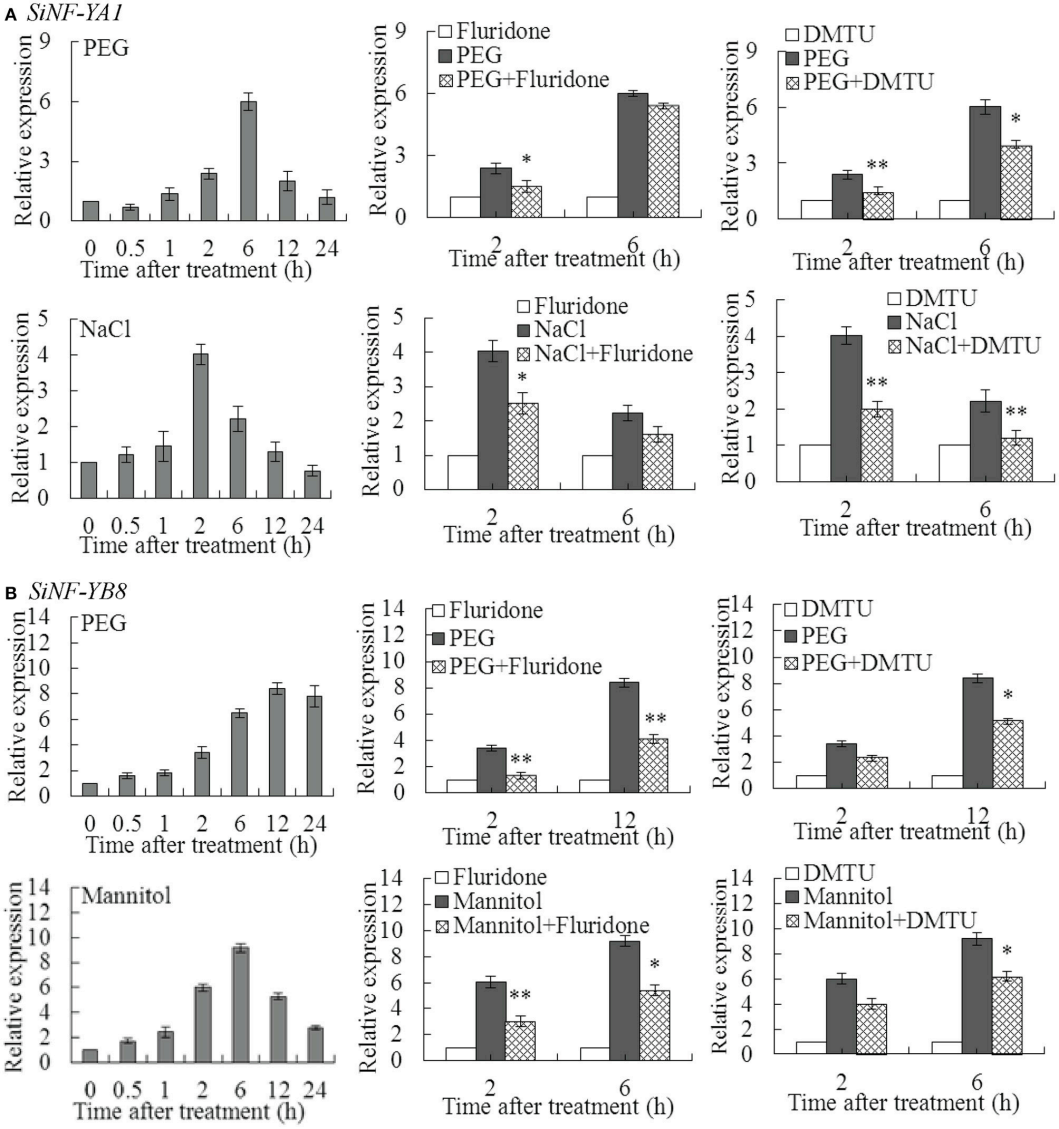
**Effects of pretreatment with inhibitor of ABA or H_2_O_2_ on *SiNF-YA1* and *SiNF-YB8* under abiotic treatments**. Effects of pretreatment with inhibitor of ABA (fluridone) or H_2_O_2_ (DMTU) on expression of **(A)** *SiNF-YA1* and **(B)** *SiNF-YB8* in foxtail millet exposed to dehydration and NaCl/mannitol. Vertical bars indicate ±SE of three replicates from one sample. In each case three biological experiments produced similar results. ^*^ and ^**^ indicate significant expression differences in comparison with the expression without inhibitor treatment at 0.01 < *P* < 0.05 and *P* < 0.01, respectively.

*SiNF-YA1* and *SiNF-YB8* were selected to perform further investigation due to relatively high up-regulated transcript levels under various stresses, respectively (Figure 5; Supplementary Table S4; Supplementary Dataset S3). *SiNF-YA1* was highly induced under PEG at 6 h (6.01 fold) and NaCl at 2 h (4.02 fold) (Figure 6A). To explore whether ABA and H_2_O_2_ were involved in up-regulation of *SiNF-YA1* under drought and salt treatments, fluridone and DMTU were chosen as the inhibitor of ABA and H_2_O_2_ (Ma et al., 2014). Treatment with fluridone and DMTU had no effect on expression of *SiNF-YA1* under drought and salt treatments (Supplementary Figure S4A). Pretreatment with the inhibitors of fluridone and DMTU prevented up-regulation of *SiNF-YA1* in PEG and NaCl treated foxtail millet seedlings (Figure 6A).

*SiNF-YB8* was highly induced under PEG at 12 h (8.41 fold) and mannitol treatment at 6 h (9.22 fold) (Figure 6B). Similar to *SiNF-YA1*, fluridone and DMTU had no effect on the expression of *SiNF-YB8* (Supplementary Figure S4B); and pretreatment with ABA and H_2_O_2_ inhibitors prevented up-regulation of *SiNF-YB8* in PEG- and mannitol-treated foxtail millet seedlings (Figure 6B). These results suggested that ABA and H_2_O_2_ were involved in up-regulation of *SiNF-YA1* and *SiNF-YB8* under PEG, NaCl or mannitol treatment, respectively.

### Subcellular localization of SiNF-YA1 and SiNF-YB8

**Figure 7:**
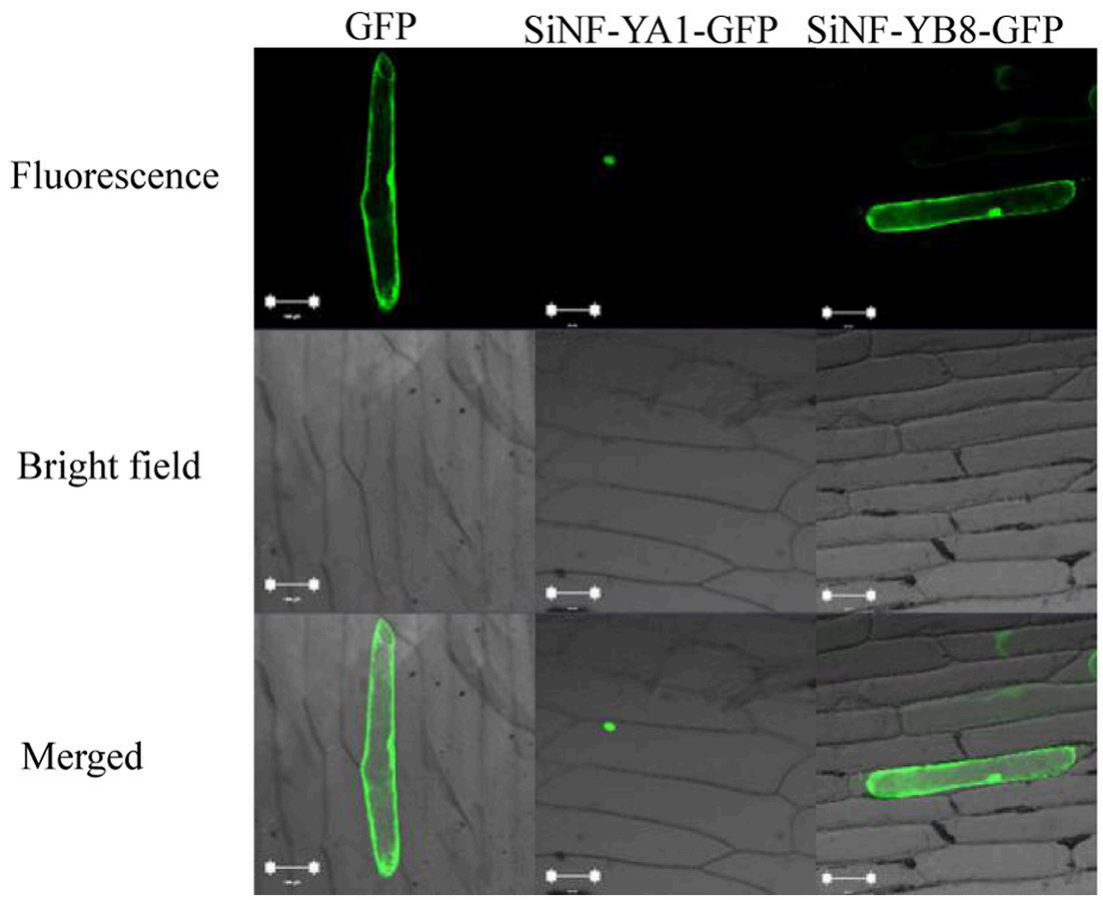
**Localization of SiNF-YA1 and SiNF-YB8 proteins**. SiNF-YA1-GFP, SiNF-YB8-GFP, and control vector (GFP) are transiently expressed in onion epidermal cells. Green fluorescence signals were detected with a laser confocal-scanning microscope. Bar, 100 μm.

Subcellular localization of a protein indicates the location at which it functions. To investigate their localization in plant cells, *SiNF-YA1* and *SiNF-YB8* were inserted into a subcellular localization vector, respectively. The recombinant vector was transformed into onion epidermal cells and observed by confocal microscopy. The GFP fluorescence of SiNF-YA1 fusion protein suggested a nuclear localization in onion epidermal cells; whereas fluorescence of SiNF-YB8 fusion protein, like the control GFP, was uniformly distributed throughout the cell, including nucleus, cytoplasm and cytomembrane (Figure 7).

### *SiNF-YA1* enhanced drought and salt tolerance in tobacco by maintaining relatively stable physiological traits

RT-PCR and qRT-PCR analysis showed that *SiNF-YA1* mRNA was expressed in all three transgenic lines but not in the wild-type (WT) plants (Supplementary Figure S5A). Under non-stressed conditions there were no differences in seed germination rate and root length between transgenic and WT plants (Supplementary Figures S6A,B). On MS medium with 3% PEG, the germination rate of WT dropped to 40.1%, whereas *35S::SiNF-YA1* transgenic seeds retained 74.3–80.1% (Supplementary Figure S6A). In a parallel experiment, the germination percentage of WT decreased to 45.0% on 50 mM NaCl media, whereas *35S::SiNF-YA1* transgenic seeds retained 80.3–91.4% (Supplementary Figures S6A,B). The growth of primary roots and young seedling leaves of *35S::SiNF-YA1* transgenic lines were indistinguishable from that of WT seedlings under normal conditions (Supplementary Figures S6A,B). When seedlings were exposed to 6% PEG for 6 d, roots of the transgenic lines were longer compared to those of WT plants (Supplementary Figures S6A,B). When seedlings were exposed to 75 mM NaCl for 6 d, growth of WT plants and transgenic lines was severely inhibited, whereas transgenic seedlings were stronger and roots of the transgenic lines were longer than those of WT plants (Supplementary Figures S6A,B). The fresh weight and dry weight of transgenic seedlings were heavier than those of WT plants (Supplementary Figure S6C).

**Figure 8:**
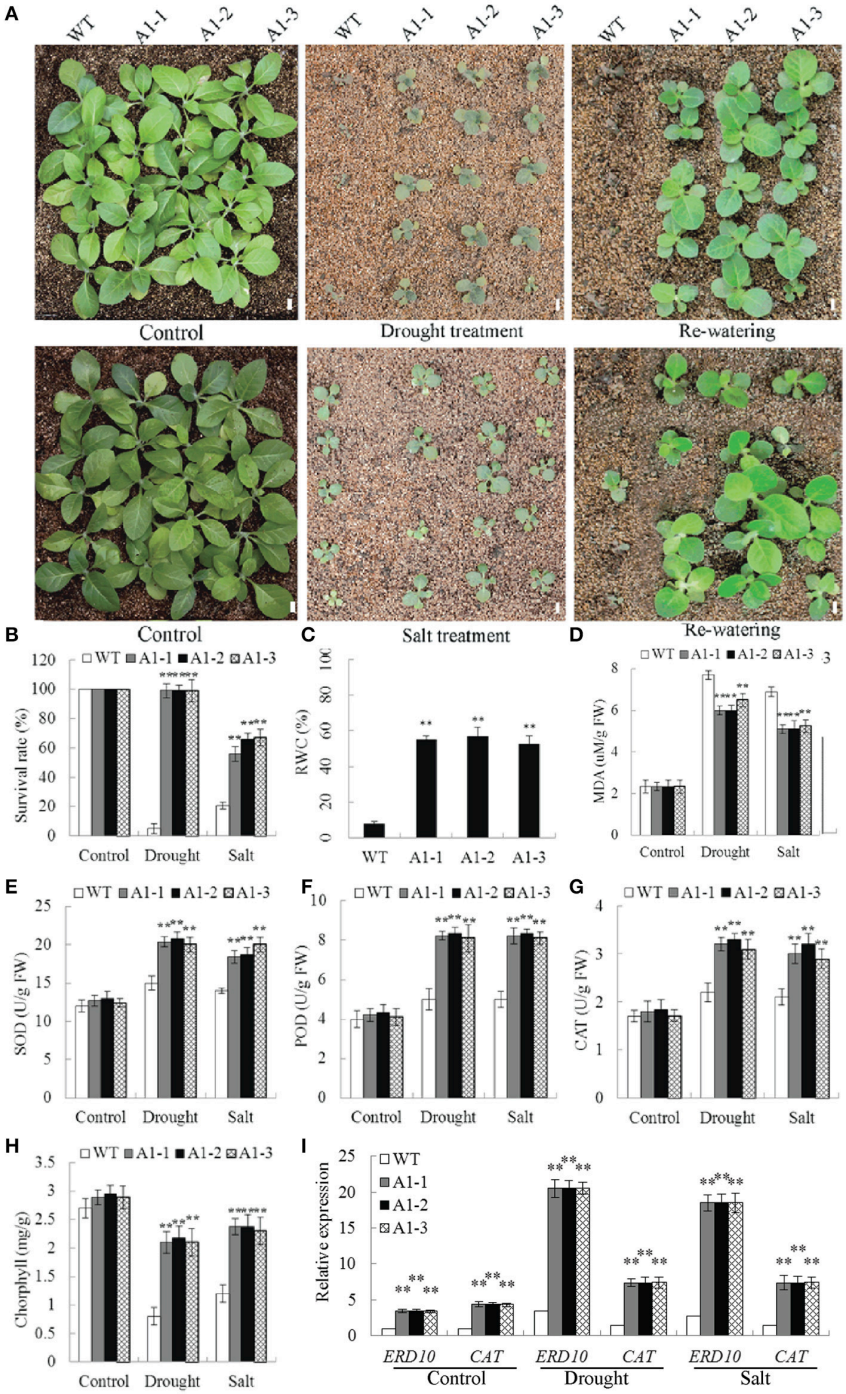
**Responses of *SiNF-YA1* transgenic tobacco to drought and salt stress in soil**. **(A)** Phenotypes of three *SiNF-YA1* transgenic lines and WT following drought and salt stress treatments. **(B–H)** Survival rates and RWC, MDA, SOD, POD, CAT, and chlorophyll contents under normal and stress conditions. **(I)** Expression analysis of stress-responsive genes in *SiNF-YA1* transgenic tobacco. Vertical bars indicate ±SE of three replicates. ^**^indicate significant differences in comparison with the WT lines at *P* < 0.01. Bar, 1 cm.

No significant difference in phenotype was observed between WT plants and *35S::SiNF-YA1* transgenic lines exposed to normal soil conditions (Figures 8A,B). After a 2-week-water-withholding treatment, the leaves of WT plants wilted severely and most became darker and died. By comparison, most of the *35S::SiNF-YA1* transgenic lines grew better than WT plants. After re-watering for 1 week, more than 95.0% of the *35S::SiNF-YA1* transgenic plants survived, whereas almost all WT seedlings were dead (Figures 8A,B). For salt treatment, WT plants and *35S::SiNF-YA1* transgenic lines were cultured for 1 week under normal conditions and then irrigated with 200 mM NaCl for 1 week. The WT plants subsequently stopped growing. However, *35S::SiNF-YA1* transgenic lines continued to grow slowly. After re-watering for 1 week, more than half of *35S::SiNF-YA1* transgenic lines remained green and continued to grow well. By comparison, most of WT plants died (Figures 8A,B). These results showed that overexpression of *SiNF-YA1* enhanced drought and salt tolerance in transgenic tobacco.

The RWC determines sensitivity to drought stress. MDA is an important indicator of membrane injury. After drought treatment, RWC in *35S::SiNF-YA1* transgenic lines was 55.7% higher than that in WT plants (Figure 8C). MDA contents in *35S::SiNF-YA1* transgenic lines and WT plants were similar under normal conditions. After drought and salt treatments, MDA content in *35S::SiNF-YA1* transgenic lines increased, but more in stressed WT plants (Figure 8D). The activities of SOD, together with POD and CAT, were higher in the transgenic lines than in WT plants under both normal and stress conditions, particularly under stress conditions (Figures 8E–G). Chlorphyll contents in *35S::SiNF-YA1* transgenic lines and WT plants were similar under normal conditions. After drought and salt treatments, chlorphyll content in *35S::SiNF-YA1* transgenic lines underwent a smaller decrease than that in WT plants (Figure 8H). These results showed that overexpression of *SiNF-YA1* enhanced drought and salt tolerances by maintaining relatively stable RWC, chlorophyll and MDA contents in *SiNF-YA1* transgenic lines under stresses.

To elucidate the possible molecular mechanisms of *SiNF-YA1* in stress responses, the expressions of a panel of stress-responsive genes were investigated in transgenic lines and WT plants under normal and stress growing conditions. qRT-PCR analysis showed that the expression level of stress responsive target genes *NtERD10* and *NtCAT* increased in *35S::SiNF-YA1* transgenic lines under both normal and stress conditions (Figure 8I).

### *SiNF-YB8* improved drought and osmotic tolerance in tobacco by improving physiological traits

RT-PCR and qRT-PCR results showed that *SiNF-YB8* mRNA was detected in all three transgenic lines but not in the WT plants (Supplementary Figure S5B). Under normal conditions, *35S::SiNF-YB8* transgenic lines showed no obvious differences with WT plants (Supplementary Figure S7). With 3% PEG, WT germination rate dropped to 20.1%, whereas the transgenic lines retained a 62.9% level. In a parallel experiment, seed germination rate of WT plants on 100 mM mannitol medium decreased to 40.7%, compared to 91.3% of *35S::SiNF-YB8* transgenic lines. When exposed to 6% PEG or 200 mM mannitol for 6 d, seedling, primary root growth, the fresh weight and dry weight of *35S::SiNF-YB8* transgenic lines was less affected than those of WT plants (Supplementary Figures S7A,B).

**Figure 9:**
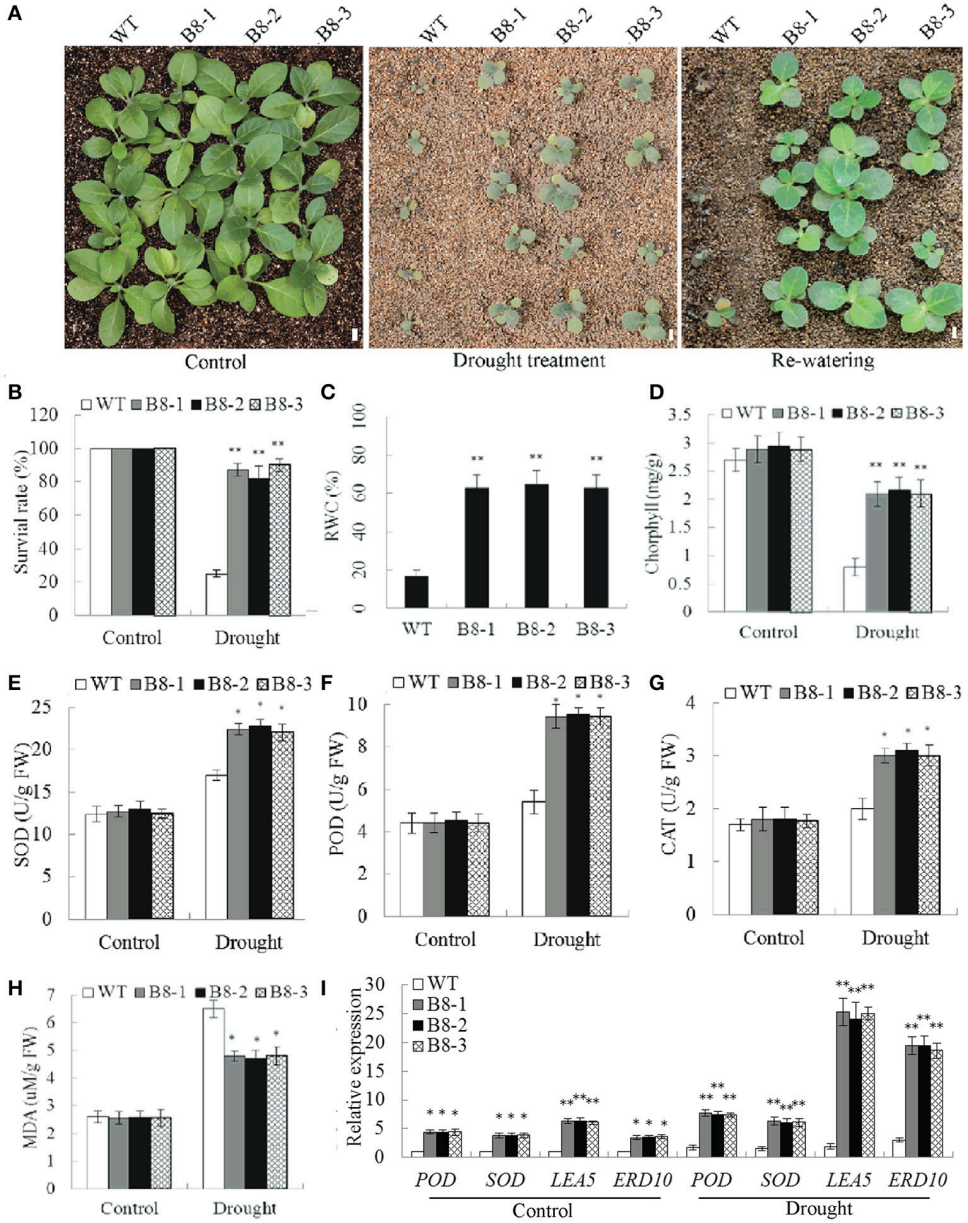
**Responses of *SiNF-YB8* transgenic tobacco to drought stress in soil. (A)** Phenotypes of the *SiNF-YB8* transgenic lines and WT following drought stress. **(B–H)** Survival rates and RWC, chlorophyll, SOD, POD, CAT, and MDA contents under normal and stress conditions. **(I)** Expression analysis of stress-responsive genes in *SiNF-YB8* transgenic tobacco. Vertical bars indicate ±SE of three replicates. ^*^, ^**^indicate significant differences in comparison with the WT lines at 0.01 < *P* < 0.05 and *P* < 0.01, respectively. Bar, 1 cm.

To examine the role of *SiNF-YB8* in drought response in soil, *35S::SiNF-YB8* transgenic lines and WT plants were withheld from water. After a 2-week water-withholding treatment, the leaves of WT plants showed wilting (Figure 9A). By comparison, *35S::SiNF-YB8* transgenic lines were slightly wilted, and grew better than WT plants (Figure 9A). After re-watering for 1 week, most of WT plants were dead, whereas more than 80.0% of *35S::SiNF-YB8* transgenic plants had survived (Figures 9A,B). Therefore, overexpression of *SiNF-YB8* enhanced drought tolerance in transgenic tobacco.

RWC, chlorphyll, and MDA contents in *35S::SiNF-YB8* transgenic lines and WT plants were similar under normal conditions. Under drought stress, RWC in *35S::SiNF-YB8* transgenic lines (65.0–65.2%) was higher than in WT plants (17.1%; Figure 9C). After drought treatment chlorophyll content in *35S::SiNF-YB8* transgenic plants declined from 2.9 to 2.1 mg/g in leaves, whereas the comparable level in WT plants dropped from 2.7 to 0.8 mg/g (Figure 9D). SOD, CAT, and POD activities were higher in the transgenic lines than in WT plants under stress conditions (Figures 9E–G). In addition, under drought stress, the MDA content of *35S::SiNF-YB8* transgenic lines underwent smaller increases than that of WT plants (Figure 9H). These results showed that overexpression of *SiNF-YB8* conferred relatively high RWC and chlorophyll contents and low MDA content, resulting in drought tolerance in transgenic lines. In addition, *SiNF-YB8* activated expression of stress-responsive genes *NtERD10, NtLEA5, NtSOD*, and *NtPOD* in tobacco under both normal and drought conditions (Figure 9I).

### SiNF-YA1 and SiNF-YB8 affected the activity of ABA- and reactive oxygen species (ROS)-responsive gene promoters

**Figure 10:**
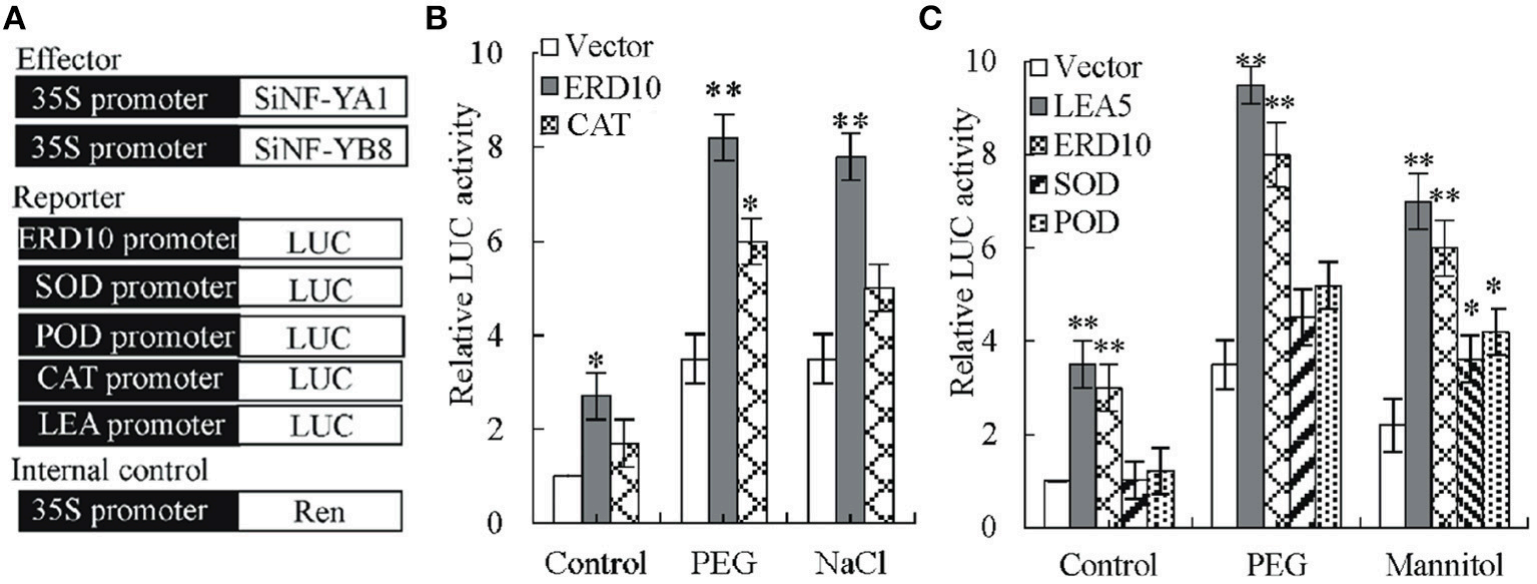
**Effects of *SiNF-YA1* and *SiNF-YB8* on the expression of *NtERD10*, *NtCAT1*, *NtSOD*, *NtPOD*, or *NtLEA5* promoters**. **(A)** Schematic diagram of different plasmid structure for transient reporter assays. **(B)** Transient reporter assays using effector *SiNF-YA1*. **(C)** Transient reporter assays using effector *SiNF-YB8*. As a negative control, empty effector vectors were used in place of *SiNF-YA1* and *SiNF-YB8* plasmids. The relative activity caused by vector control was set as 1. Vertical bars indicate ± SE of three replicates. ^*^, ^**^ indicate significant differences in comparison with the relative LUC activity in negative control at 0.01 < *P* < 0.05 and *P* < 0.01, respectively.

Transient luciferase (LUC) assays in tobacco protoplast were used to determine SiNF-YA1 activity against tobacco *NtERD10* and *NtCAT* promoters (Figure 10A; Supplementary Dataset S4). The relative luciferase activity (Luc/Ren) was noticeably enhanced when transformation was performed with SiNF-YA1 effector against *NtERD10* promoter (Luc/Ren 2.7), compared with the no-effector control (Luc/Ren 1) (Figure 10B). Under stress treatments, activities of luciferase against *NtERD10* and *NtCAT* promoter increased (Figure 10B). However, there were no detectable obvious activities of luciferase against *NtCAT* promoter under salt stress treatments.

Similar to SiNF-YA1, transient luciferase assays in tobacco protoplast were also used to determine SiNF-YB8 activity against tobacco *NtERD10, NtSOD, NtPOD*, and *NtLEA5* promoters (Figure 10A; Supplementary Dataset S4). The relative luciferase activity (Luc/Ren) was noticeably enhanced when transformation was performed with SiNF-YB8 effector against *NtERD10* and *NtLEA5* promoters (Luc/Ren 3.0 and 3.5, respectively) under normal conditions (Figure 10C), while no obvious activity changes were detected against *NtPOD* and *NtSOD* promoters. Under PEG stress conditions, only luciferase activities against *NtERD10* and *NtLEA5* increased (Figure 10C). Under mannitol stress conditions, all luciferase activities increased (Figure 10C).

## Discussion

Previous studies demonstrated that NF-Y transcription factor genes played important roles in abiotic stress (Nelson et al., 2007; Stephenson et al., 2007; Han et al., 2013; Ni et al., 2013). It provided an impetus for investigation of the biological roles of NF-Y family genes in foxtail millet, an ideal cereal crop suited for study of abiotic stress tolerance (Doust et al., 2009; Brutnell et al., 2010; Zhang et al., 2012; Lata et al., 2013).

### Characterization of NF-Ys in foxtail millet

In the present study, a total of 39 NF-Ys from foxtail millet were identified. Multiple alignments showed NF-YA proteins might be more evolutionarily constrained than either NF-YB or NF-YC proteins (Figure 1). Therefore, it was speculated that specific functions were highly conserved in essentially all NF-YA proteins. NF-YA subunits might act as a strong constraint against post duplication evolutionary divergence while the NF-YB/NF-YC subunits might be responsible for diverse interactions that rapidly diversified in the plant lineage. The conserved motifs in NF-Y proteins among different plant species suggested that some motifs might be unique to monocots (Supplementary Figure S1). Previous studies focused on conserved NF-Y core domains, while these unique motifs might play more important roles in evolution process. Synteny analysis showed that some SiNF-Y genes were involved in syntenic blocks (Figure 4), indicating that tandem and segmental duplication events play major roles in the expansion of the NF-Ys in foxtail millet. The estimated Ka/Ks for NF-Y gene pairs were under strong purifying selection pressure.

Among NF-Y transcription factors, NF-YB subunits did not have nuclear localization signal (NLS) and the subcellular localization of NF-YB might be influenced by the availability of NF-YC or post-modification. However, NF-YA and NF-YC subunits could be imported into the nucleus without auxiliary assistance. For example, *Arabidopsis* NF-YB3 and NF-YB10 were localized to the cytoplasm under normal conditions. Under stress-treated conditions, AtNF-YB3 could be relocated to the nucleus. When AtNF-YB10 interacted with AtNF-YC2, AtNF-YB10 could be transported into the nucleus (Liu and Howell, 2010; Hackenberg et al., 2012b). However, PdNF-YB7, OsHAP3H (OsDTH8), and AtNF-YB2 protein were localized to the nucleus, respectively (Liu and Howell, 2010; Wei et al., 2010; Hackenberg et al., 2012b; Han et al., 2013). Subcellular localization showed that SiNF-YA1 was localized to the nucleus and SiNF-YB8 has a cell-wide distribution pattern (Figure 7). Further experiments are now needed to elucidate the translocation mechanism of NF-Ys which will directly influence NF-Y transcriptional activity.

### Evolution of NF-Ys implied potential functions

Previous genomes examination suggested high correlation and high sequence homology within the same NF-Y subunit family resulted in partially redundancy or overlapping functionality, which may be beneficial for protecting the cell from various stress conditions and selecting candidate genes for agriculture production. For NF-YA subunits, SiNF-YA5 was the best orthology match of rice OsHAP2E (namely OsNF-YA8; Figure 2A). Overexpression of *OsHAP2E* conferred resistance to pathogens, salinity and drought, and increased photosynthesis and tiller number in rice (Alam et al., 2015). Recently, it was demonstrated that NF-YA genes were post-transcriptionally regulated by *microRNA169* (*miR169*), suggesting a complex mechanism controlling the functions of NF-YA genes (Zhao et al., 2009; Han et al., 2013; Ni et al., 2013; Sorin et al., 2014). *OsHAP2E* was identified as a target gene of *miR-169g* and *miR-169n* (*o*) (Liu and Howell, 2010). Based on the conservation of miR169 in plants, it was likely that a similar mechanism controlled the accumulation of NF-YA mRNA in foxtail millet. For NF-YB subunits, SiNF-YB6 and SiNF-YB9 were the best orthology matches of LEC1-type proteins *Arabidopsis* LEC1 (AtNF-YB9) and L1L (AtNF-YB6) that were involved in embryogenesis, seed or silique development (Figures 2B, 4C; Lotan et al., 1998; Kwong et al., 2003; Warpeha et al., 2007; Yamamoto et al., 2009). These pairs of LEC1-type proteins may share similar functions (Cagliari et al., 2014). SiNF-YB2, SiNF-YB3 and SiNF-YB11 were the best orthology matches to rice OsHAP3A, OsHAP3B, and OsHAP3C belonging to group D (Figures 2B, 4C). OsHAP3 had been identified to be involved in seed maturation and chloroplast biogenesis (Miyoshi et al., 2003). *SiNF-YB2* and *SiNF-YB11* were also orthologous to *AtNF-YB1* that improved performance under drought conditions in *Arabidopsis* (Nelson et al., 2007). *SiNF-YB3* was orthologous to *TaNF-YB2* that was significantly up-regulated in response to drought in wheat (Stephenson et al., 2007). *SiNF-YB7* belonging to group E was orthologous to *OsHAP3H* which regulated grain productivity, plant height and heading date in rice (Figures 2B, 4C; Wei et al., 2010). SiNF-YB4 was orthologous to AtNF-YB3 and TaNF-YB3. *AtNF-YB3* had been identified as a photoperiod-dependent flowering time, ER and drought stress regulator (Kumimoto et al., 2008; Liu and Howell, 2010). TaNF-YB3 was identified as a target for photosynthesis based on microarray data (Stephenson et al., 2011). Recent evidence suggested that *AtNF-YC3* and *AtNF-YC9* were involved in control of flowering and *AtNF-YC2* was involved in endoplasmic reticulum (ER) stress (Liu and Howell, 2010; Stephenson et al., 2010). The best orthology match of SiNF-YC5 protein belonging to group F was TaNF-YC11 which was involved in the regulation of photosynthesis genes (Figure 2C; Hackenberg et al., 2012a). Therefore, most of NF-YA/B/C subunits were involved in the abiotic stress responses or development.

### NF-Ys were involved in ABA and ROS-mediated abiotic stress responses

Past studies mainly focused on the functions of NF-Ys in plant development (Zhang et al., 2002; Yazawa and Kamada, 2007; Combier et al., 2008; Stephenson et al., 2010; Cao et al., 2011, 2014; Liang et al., 2012; Li et al., 2013; Mu et al., 2013; Hilioti et al., 2014; Laloum et al., 2014), whereas studies on their functions in abiotic stress were relatively limited. In the present work, we analyzed responses of 11 up-regulated SiNF-Y genes to abiotic stresses in different tissues (Figure 5). The results implied that most NF-Y members might be putative regulators of response to abiotic stress. Specific expression profiles in specific tissues indicated that the expansion of NF-Y families in plants resulted in sub-functionalization of some subunit members.

Drought and salt stresses often result from imbalance between ROS producting and ROS scavenging ability that cause damage to various cellular components, such as carbohydrates, lipids, proteins and nucleic acids (Selote and Khanna-Chopra, 2006; Cagliari et al., 2014). Many studies showed that ROS signaling was one of the key mechanisms involved in drought and salt tolerance, and the maintenance of the balance between ROS production and ROS scavenging was essential for drought and salt tolerance in plants. ROS enhancement under stress acts as an alarm signal that triggered acclimatory/defense responses by specific signal transduction pathways, which might be linked to ABA, Ca^2+^ fluxes and sugar sensing occurring both upstream and downstream of the ABA-dependent signaling pathways. The NF-Y complex coordinates oxidative stress response in eukaryotes (Hackenberg et al., 2012a; Ikbal et al., 2014). Pretreatment with H_2_O_2_ inhibitor prevented up-regulation of *SiNF-YA1* and *SiNF-YB8* in dehydration-, NaCl-, and/or mannitol-treated foxtail millet seedlings (Figure 6). Thus, it is plausible that *SiNF-YA1* and *SiNF-YB8* could be part of the ROS-mediation processes. Recently, it was reported that transcripts of rice *HAP2E* and bermuda grass *NF-YC1* in response to drought and salinity stresses might be involved in H_2_O_2_ signaling pathway (Thön et al., 2010; Chen et al., 2015). However, more research is needed to confirm this mechanism.

### NF-Ys improved stress tolerance by mediating ABA-dependent and ROS signal pathways

Many transcription factors responding to stress have been identified, providing an insight that plants develop flexible molecular and cellular mechanisms to tolerate abiotic stress (Xu et al., 2008, 2011). Transcripts of ABA- dependent and independent pathway markers increased in polar *NF-YB7* and bermuda grass *NF-YC1* transgenic plants (Han et al., 2013; Chen et al., 2015). In addition, it was found that coexpression of *PpABI3A* along with *PpNF-YC1* and *PpNF-YB4* synergistically activated *PpLEA1* promoter through the ACTT-core ABRE element in *Physcomitrella patens* (Yotsui et al., 2013). Our results showed that *NtERD10* and *NtLEA5*, ABA-dependent LEA genes, were strongly up-regulated in *SiNF-YA1* or *SiNF-YB8* transgenic tobacco (Figures 8I, 9I; Wu et al., 2008), and many ABRE *cis*-acting elements were found in promoters of these two tobacco LEA genes (Table 2). This might imply foxtail millet *SiNF-YA1* and *SiNF-YB8* were involved in ABA-dependent stress signal pathway.

**Table 2:** **Promoter sequence analysis of genes up-regulated by *SiNF-YA1* or *SiNF-YB8* in transgenic tobacco**.

**Gene name**	**Locus name**	**CCAAT-box**	**ABRE-box**
*NtERD10C*	AB049337.1	4	9
*NtLEA5*	AF053076.1	2	22
*NtSOD*	AB093097.1	1	14
*NtPOD*	U15933.1	1	12
*NtCAT*	U93244.1	3	14

In addition, *SiNF-YA1* and *SiNF-YB8* up-regulated oxidative stress-responsive genes, such as *NtCAT, NtSOD* or *NtPOD* (Figures 8I, 9I). CCAAT *cis*-acting elements were found in promoters of these oxidative stress-responsive genes (Table 2). The transient expression assay showed that *SiNF-YA1* and *SiNF-YB8* could activate expression of oxidative stress-related genes under stress conditions (Figure 10). It was previously shown that overexpression of *AtNF-YA5* was likely important for dehydration tolerance *via* its role in activating oxidative stress-responsive genes (Li et al., 2008). Physiological and biochemical analysis also showed *SiNF-YA1* or *SiNF-YB8* enhanced stress tolerance through enhancing the antioxidant system (Figures 8E–G, 9E–G). Recent reports revealed that the NF-Y heterotrimer was regulated by the redox status of the cell serving for a coordinated activation and deactivation of antioxidant defense mechanisms, including the specific transcriptional activator NapA, production of enzymes such as CAT, thioredoxin or POD, and maintenance of a distinct glutathione (ASH) homeostasis (Ikbal et al., 2014). As several other transcription factors (Yoshioka et al., 2006), the antioxidant system might be involved in the activity of NF-Y to confer abiotic stress tolerance in plants.

In conclusion, we proposed foxtail millet NF-YA or NF-YB acted as key component of stress tolerance by being involved in both ABA-dependent and ROS signal pathways.

## Author contributions

ZX coordinated the project, conceived and designed experiments, and edited the manuscript. ZF conducted the bioinformatic work, generated and analyzed data, and wrote the manuscript. GH performed experiments and analyzed the data. PL, WZ, and MC provided analytical tools. YG and YM contributed with valuable discussions. All authors have read and approved the final manuscript.

### Conflict of interest statement

The authors declare that the research was conducted in the absence of any commercial or financial relationships that could be construed as a potential conflict of interest. The reviewer Chenghao Li declares that, despite being affiliated with the same institute as the author Wei-Jun Zheng, the review process was carried out objectively.

## Supplementary material

Supplementary Dataset S1 **Fasta file of all foxtail millet NF-Y protein sequences**.

Supplementary Dataset S2 **Fasta file of all foxtail millet NF-Y protein conserved regions**.

Supplementary Dataset S3 **CDS and protein sequences of SiNF-YA1 and SiNF-YB8**.

Supplementary Dataset S4 **Fasta file of tobacco *ERD10, CAT, SOD, POD*, and *LEA5* promoters**.

Supplementary Figure S1 **Motifs identified for NF-Y proteins from foxtail millet, *Arabidopsis*, wheat, rice, and *Brachypodium* using MEME motif search tool**. Motif structures for **(A)** NF-YA; **(B)** NF-YB; **(C)** NF-YC proteins. Different motifs are shown as differently colored boxes.

Supplementary Figure S2 **Conserved motifs logos identified for the NF-Y proteins from foxtail millet, *Arabidopsis*, wheat, rice, and *Brachypodium* by MEME software**.

Supplementary Figure S3 **Chromosomal locations of 39 SiNF-Y genes in foxtail millet**. The chromosome numbers are indicated at the top of each bar.

Supplementary Figure S4 **Expression patterns of *SiNF-YA1* and *SiNF-YB8* under fluridone or DMTU treatment. (A)** *SiNF-YA1* gene; **(B)** *SiNF-YB8* gene. Vertical bars indicate ± SE of three replicates from one sample. Three biological experiments were performed, and produced similar results.

Supplementary Figure S5 **Expression patterns of *SiNF-YA1* and *SiNF-YB8* in transgenic tobacco. (A)** Expression of *SiNF-YA1* gene detected by RT-PCR and qRT-PCR; **(B)** Expression of *SiNF-YB8* gene detected by RT-PCR and qRT-PCR. The expression level of *SiNF-YA1*/*SiNF-YB8* in transgenic tobacco A1-1/B8-1 was seen as 1.

Supplementary Figure S6 **Overexpression of *SiNF-YA1* enhanced tolerance to drought and salt stresses during germination and seedling growth stages in transgenic tobacco. (A)** Germination rates and root lengths of *SiNF-YA1* transgenic plants grown on MS media containing PEG or NaCl. **(B)** Mean germination rates and root lengths. **(C)** Fresh weigh and dry weight. A1-1, A1-2, A1-3: *35S::SiNF-YA1* transgenic lines; WT: wild type. Vertical bars indicate ± SE of three replicates. ^**^ indicate significant differences in comparison with the WT lines at *P* < 0.01. Bar, 1 cm.

Supplementary Figure S7 **Overexpression of *SiNF-YB8* enhanced tolerance to drought and osmotic stresses during germination and seedling growth stages in transgenic tobacco. (A)** Germination and root lengths of *SiNF-YB8* transgenic plants were tested on MS medium containing PEG and mannitol. The seeds were allowed to grow for 6 d when the growth patterns of roots were monitored and photographed. **(B)** Mean germination rates and root lengths were analyzed. **(C)** Fresh weigh and dry weight. B8-1, B8-2, B8-3: *35S::SiNF-YB8* transgenic lines. Vertical bars indicate ± SE of three replicates. ^**^ indicate significant differences in comparison with the WT lines at *P* < 0.01. Bar, 1 cm.

Supplementary Table S1 **Primers used in this text**.

Supplementary Table S2 **Conserved motifs identified for (A) NF-YA, (B) NF-YB, and (C) NF-YC proteins by MEME software**.

Supplementary Table S3 **The Ka/Ks ratio and % identity for orthologous SiNF-Ys with other plant NF-Ys**.

Supplementary Table S4 **Drought-induced foxtail millet NF-Y genes from the *de novo* transcriptome assembly of foxtail millet sequencing data**. FDR, false discovery rate.

## Abbreviations

ABA: abscisic acid
CAT: catalase
DMTU: dimethyl thiourea
GFP: green fluorescent protein
LUC: luciferase
MDA: malondialdehyde
NF-Y: Nuclear Factor Y
ORF: open reading frame
POD: peroxidase
qRT-PCR: quantitative RT-PCR
ROS: reactive oxygen species
RWC: relative water content
SOD: superoxide dismutase
Tail-PCR: thermal asymmetric inter-laced PCR
WT: wild-type.

## References

[B1] Alam M. M. Tanaka T. Nakamura H. Ichikawa H. Kobayashi K. Yaeno T.. (2015). Overexpression of a rice heme activator protein gene *OsHAP2E* confers resistance to pathogens, salinity, drought, increases photosynthesis, tiller number. Plant Biotechnol. J. 13, 85–96. 10.1111/pbi.12239 25168932

[B2] Brutnell T. P. Wang L. Swartwood K. Goldschmidt A. Jackson D. Zhu X. G.. (2010). *Setaria viridis*, a model for C4 photosynthesis. Plant Cell 22, 2537–2544. 10.1105/tpc.110.075309 20693355 PMC2947182

[B3] Cagliari A. Turchetto-Zolet A. C. Korbes A. P. Maraschin Fdos S. Margis R. Margis-Pinheiro M. (2014). New insights on the evolution of Leafy cotyledon1 LEC1 type genes in vascular plants. Genomics 103, 380–387. 10.1016/j.ygeno.2014.03.005 24704532

[B4] Cao S. Kumimoto R. W. Siriwardana C. L. Risinger J. R. Holt B. F. III. (2011). Identification, characterization of NF-Y transcription factor families in the monocot model plant *Brachypodium distachyon*. PLoS ONE 6:e21805. 10.1371/journal.pone.0021805 21738795 PMC3128097

[B5] Cao S. Kumimoto R. W. Gnesutta N. Calogero A. M. Mantovani R. Holt B. F. III. (2014). A distal CCAAT/NUCLEAR FACTOR Y complex promotes chromatin looping at the *FLOWERING LOCUS T* promoter, regulates the timing of flowering in *Arabidopsis*. Plant Cell 26, 1009–1017. 10.1105/tpc.113.120352 24610724 PMC4001365

[B6] Chen M. Zhao Y. Zhuo C. Lu S. Guo Z. (2015). Overexpression of a NF-YC transcription factor from bermudagrass confers tolerance to drought, salinity in transgenic rice. Plant Biotechnol. J. 13, 482–491. 10.1111/pbi.12270 25283804

[B7] Combier J. P. de Billy F. Gamas P. Niebel A. Rivas S. (2008). Trans-regulation of the expression of the transcription factor MtHAP2-1 by a uORF controls root nodule development. Genes Dev. 22, 1549–1559. 10.1101/gad.461808 18519645 PMC2418590

[B8] Dorn A. Bollekens J. Staub A. Benoist C. Mathis D. (1997). A multiplicity of CCAAT box-binding proteins. Cell 50, 863–872. 10.1016/0092-8674(87)90513-7 3476205

[B9] Doust A. N. Kellogg E. A. Devos K. M. Bennetzen J. L. (2009). Foxtail millet, a sequence-driven grass model system. Plant Physiol. 149, 137–141. 10.1104/pp.108.129627 19126705 PMC2613750

[B10] Edwards D. Murray J. A. Smith A. G. (1998). Multiple genes encoding the conserved CCAAT-box transcription factor complex are expressed in *Arabidopsis*. Plant Physiol. 117, 1015–1022. 10.1104/pp.117.3.1015 9662544 PMC34917

[B11] FitzGerald P. C. Shlyakhtenko A. Mir A. A. Vinson C. (2004).Clustering of DNA sequences in human promoters. Genome Res. 41, 1562–1574. 10.1101/gr.1953904 15256515 PMC509265

[B12] Frontini M. Imbriano C. Manni I. Mantovani R. (2004). Cell cycle regulation of NF-YC nuclear localization. Cell Cycle 3, 217–222. 10.4161/cc.3.2.654 14712092

[B13] Hackenberg D. Keetman U. Grimm B. (2012a). Homologous *NF-YC2* subunit from *Arabidopsis*, tobacco is activated by photooxidative stress and induces flowering. Int. J. Mol. Sci. 13, 3458–3477. 10.3390/ijms13033458 22489162 PMC3317722

[B14] Hackenberg D. Wu Y. Voigt A. Adams R. Schramm P. Grimm B. (2012b). Studies on differential nuclear translocation mechanism, assembly of the three subunits of the *Arabidopsis thaliana* transcription factor NF-Y. Mol. Plant 5, 876–888. 10.1093/mp/ssr107 22199235

[B15] Han X. Tang S. An Y. Zheng D. C. Xia X. L. Yin W. L. (2013). Overexpression of the poplar *NF-YB7* transcription factor confers drought tolerance, improves water-use efficiency in *Arabidopsis*. J. Exp. Bot. 64, 4589–4601. 10.1093/jxb/ert262 24006421 PMC3808328

[B16] He H. Dong Q. Shao Y. Jiang H. Zhu S. Cheng B.. (2012). Genome-wide survey, characterization of the WRKY gene family in *Populus trichocarpa*. Plant Cell Rep. 31, 1199–1217. 10.1007/s00299-012-1241-0 22371255

[B17] Hilioti Z. Ganopoulos I. Bossis I. Tsaftaris A. (2014). LEC1-LIKE paralog transcription factor, how to survive extinction fit in NF-Y protein complex. Gene 543, 220–233. 10.1016/j.gene.2014.04.019 24727055

[B18] Hu W. Huang C. Deng X. Zhou S. Chen L. Li Y.. (2013). *TaASR1*, a transcription factor gene in wheat, confers drought stress tolerance in transgenic tobacco. Plant Cell Environ. 36, 1449–1464. 10.1111/pce.12074 23356734

[B19] Huang W. Sun W. Lv H. Luo M. Zeng S. Pattanaik S. (2013). A R2R3-MYB transcription factor from *Epimedium sagittatum* regulates the flavonoid biosynthetic pathway. PLoS ONE 1:e70778 10.1371/journal.pone.0070778 23936468 PMC3731294

[B20] Ikbal F. E. Hernández J. A. Barba-Espín G. Koussa T. Aziz A. Faize M.. (2014). Enhanced salt-induced antioxidative responses involve a contribution of polyamine biosynthesis in grapevine plants. J. Plant Physiol. 171, 779–788. 10.1016/j.jplph.2014.02.006 24877669

[B21] Keddie J. Adam L. Pineda O. Ratcliffe O. J. Samaha R. R. Creelman R.. (2000). *Arabidopsis* transcription factors, genome-wide comparative analysis among eukaryotes. Science 290, 2105–2110. 10.1126/science.290.5499.2105 11118137

[B22] Kumimoto R. W. Adam L. Hymus G. J. Repetti P. P. Reuber T. L. Marion C. M.. (2008). The nuclear factor Y subunits *NF-YB2, NF-YB3* play additive roles in the promotion of flowering by inductive long-day photoperiods in *Arabidopsis*. Planta 228, 709–723. 10.1007/s00425-008-0773-6 18600346

[B23] Kwong R. W. Bui A. Q. Lee H. Kwong L. W. Fischer R. L. Goldberg R. B.. (2003). *LEAFY COTYLEDON1-LIKE* defines a class of regulators essential for embryo development. Plant Cell 15, 5–18. 10.1105/tpc.006973 12509518 PMC143447

[B24] Laloum T. Baudin M. Frances L. Lepage A. Billault-Penneteau B. Cerri M. R.. (2014).Two CCAAT box-binding transcription factors redundantly regulate early steps of the legume-rhizobia endosymbiosis. Plant J. 79, 757–768. 10.1111/tpj.12587 24930743

[B25] Lata C. Gupta S. Prasad M. (2013). Foxtail millet, a model crop for genetic, genomic studies in bioenergy grasses. Crit. Rev. Biotechnol. 33, 328–343. 10.3109/07388551.2012.716809 22985089

[B26] Li W. X. Oono Y. Zhu J. He X. J. Wu J. M. Iida K. (2008). The *Arabidopsis NFYA5* transcription factor is regulated transcriptionally, post-transcriptionally to promote drought resistance. Plant Cell 20, 2238–2251. 10.1105/tpc.108.059444 18682547 PMC2553615

[B27] Li Y. J. Fang Y. Fu Y. R. Huang J. G. Wu C. A. Zheng C. C. (2013). *NFYA1* is involved in regulation of post germination growth arrest under salt stress in *Arabidopsis*. PLoS ONE 8:e61289. 10.1371/journal.pone.0061289 23637805 PMC3634844

[B28] Liang M. Hole D. Wu J. Blake T. Wu Y. (2012). Expression, functional analysis of NUCLEAR FACTOR-Y, subunit B genes in barley. Planta 235, 779–791. 10.1007/s00425-011-1539-0 22042327

[B29] Liu J. X. Howell S. H. (2010). bZIP28, NF-Y transcription factors are activated by ER stress, assemble into a transcriptional complex to regulate stress response genes in *Arabidopsis*. Plant Cell 22, 782–796. 10.1105/tpc.109.072173 20207753 PMC2861475

[B30] Liu P. Xu Z. S. Pan P. L. Hu D. Chen M. Li L. C.. (2013). A wheat *PI4K* gene whose product possesses threonine autophophorylation activity confers tolerance to drought, salt in *Arabidopsis*. J. Exp. Bot. 64, 2915–2927. 10.1093/jxb/ert133 23682116 PMC3741686

[B31] Lotan T. Ohto M. Yee K. M. West M. A. Lo R. Kwong R. W.. (1998). *Arabidopsis LEAFY COTYLEDON1* is sufficient to induce embryo development in vegetative cells. Cell 93, 1195–1205. 10.1016/S0092-8674(00)81463-4 9657152

[B32] Ma F. Wang L. Li J. Samma M. K. Xie Y. Wang R.. (2014). Interaction between HY1, H_2_O_2_ in auxin-induced lateral root formation in *Arabidopsis*. Plant Mol. Biol. 85, 49–61. 10.1007/s11103-013-0168-3 24366686

[B33] Maity S. N. de Crombrugghe B. (1992).Biochemical analysis of the B subunit of the heteromeric CCAT-binding factor. J. Biol. Chem. 267, 8286–8292. 1569083

[B34] Mantovani R. (1999). The molecular biology of the CCAAT-binding factor NF-Y. Gene. 239, 15–27. 10.1016/S0378-1119(99)00368-6 10571030

[B35] Miyoshi K. Ito Y. Serizawa A. Kurata N. (2003). *OsHAP3* genes regulate chloroplast biogenesis in rice. Plant J. 36, 532–540. 10.1046/j.1365-313X.2003.01897.x 14617083

[B36] Mu J. Tan H. Hong S. Liang Y. Zuo J. (2013). *Arabidopsis* transcription factor genes *NF-YA1, 5, 6, 9* play redundant roles in male gametogenesis, embryogenesis, seed development. Mol. Plant 6, 188–201. 10.1093/mp/sss061 22933713

[B37] Nelson D. E. Repetti P. P. Adams T. R. Creelman R. A. Wu J. Warner D. C.. (2007). Plant nuclear factor Y NF-Y B subunits confer drought tolerance, lead to improved corn yields on water-limited acres. Proc. Natl. Acad. Sci. U.S.A. 104, 16450–16455. 10.1073/pnas.0707193104 17923671 PMC2034233

[B38] Ni Z. Hu Z. Jiang Q. Zhang H. (2013). *GmNFYA3*, a target gene of *miR169*, is a positive regulator of plant tolerance to drought stress. Plant Mol. Biol. 82, 113–129. 10.1007/s11103-013-0040-5 23483290

[B39] Puranik S. Jha S. Srivastava P. S. Sreenivasulu N. Prasad M. (2011). Comparative transcriptome analysis of contrasting foxtail millet cultivars in response to short-term salinity stress. J. Plant Physiol. 168, 280–287. 10.1016/j.jplph.2010.07.005 20708821

[B40] Quevillon E. Silventoinen V. Pillai S. Harte N. Mulder N. Apweiler R.. (2005). InterProScan, protein domains identifier. Nucleic Acids Res. 33, W116–W120. 10.1093/nar/gki442 15980438 PMC1160203

[B41] Romier C. Cocchiarella F. Mantovani R. Moras D. (2003). The NF-YB/NF-YC structure gives insight into DNA binding, transcription regulation by CCAAT factor NF-Y. J. Biol. Chem. 278, 1336–1345. 10.1074/jbc.M209635200 12401788

[B42] Selote D. S. Khanna-Chopra R. (2006). Drought acclimation confers oxidative stress tolerance by inducing co-ordinated antioxidant defense at cellular, subcellular level in leaves of wheat seedlings. Plant Physiol. 127, 494–506. 10.1111/j.1399-3054.2006.00678.x

[B43] Siefers N. Dang K. K. Kumimoto R. W. Bynum W. E. IV. Tayrose G. Holt B. F. III. (2009). Tissue-specific expression patterns of *Arabidopsis* NF-Y transcription factors suggest potential for extensive combinatorial complexity. Plant Physiol. 149, 625–641. 10.1104/pp.108.130591 19019982 PMC2633833

[B44] Sinha S. Maity S. N. Lu J. de Crombrugghe B. (1995). Recombinant rat CBF-C, the third subunit of CBF/NFY, allows formation of a protein-DNA complex with CBF-A, CBF-B, with yeast HAP2, HAP3. Proc. Natl. Acad. Sci. U.S.A. 92, 1624–1628. 10.1073/pnas.92.5.1624 7878029 PMC42572

[B45] Sorin C. Declerck M. Christ A. Blein T. Ma L. Lelandais-Brière C.. (2014). A miR169 isoform regulates specific NF-YA targets, root architecture in *Arabidopsis*. New Phytol. 202, 1197–1211. 10.1111/nph.12735 24533947

[B46] Stephenson T. J. McIntyre C. L. Collet C. Xue G. P. (2007). Genome-wide identification, expression analysis of the NF-Y family of transcription factors in *Triticum aestivum*. Plant Mol. Biol. 65, 77–92. 10.1007/s11103-007-9200-9 17598077

[B47] Stephenson T. J. McIntyre C. L. Collet C. Xue G. P. (2010). *TaNF-YC11*, one of the light-upregulated NF-YC members in *Triticum aestivum*, is co-regulated with photosynthesis-related genes. Funct. Integr. Genomics 10, 265–276. 10.1007/s10142-010-0158-3 20111976

[B48] Stephenson T. J. McIntyre C. L. Collet C. Xue G. P. (2011). *TaNF-YB3* is involved in the regulation of photosynthesis genes in *Triticum aestivum*. Funct. Integr. Genomics 11, 327–340. 10.1007/s10142-011-0212-9 21327447

[B49] Tamura K. Dudley J. Nei M. Kumar S. (2007). MEGA4, molecular evolutionary genetics analysis MEGA software version 4.0. Mol. Biol. Evol. 24, 1596–1599 10.1093/molbev/msm092 17488738

[B50] Testa A. Donati G. Yan P. Romani F. Huang T. H. Viganò M. A.. (2005). Chromatin immunoprecipitation ChIP on chip experiments uncover a widespread distribution of NF-Y binding CCAAT sites outside of core promoters. J. Biol. Chem. 280, 13606–13615. 10.1074/jbc.M414039200 15647281

[B51] Thirumurugan T. Ito Y. Kubo T. Serizawa A. Kurata N. (2008). Identification, characterization, interaction of HAP family genes in rice. Mol. Genet. Genomics 279, 279–289. 10.1007/s00438-007-0312-3 18193457

[B52] Thompson J. D. Gibson T. J. Plewniak F. Jeanmougin F. Higgins D. G. (1997). The CLUSTAL X interface, flexible strategies for multiple sequence alignment aided by quality analysis tools. Nucleic Acids Res. 25, 4876–4882. 10.1093/nar/25.24.4876 9396791 PMC147148

[B53] Thön M. Al Abdallah Q. Hortschansky P. Scharf D. H. Eisendle M. Haas H.. (2010). The CCAAT-binding complex coordinates the oxidative stress response in eukaryotes. Nucleic Acids Res. 38, 1098–1113. 10.1093/nar/gkp1091 19965775 PMC2831313

[B54] Warpeha K. M. Upadhyay S. Yeh J. Adamiak J. Hawkins S. I. Lapik Y. R.. (2007). The GCR1, GPA1, PRN1, NF-Y signal chain mediates both blue light, abscisic acid responses in *Arabidopsis*. Plant Physiol. 143, 1590–1600. 10.1104/pp.106.089904 17322342 PMC1851835

[B55] Wei X. Xu J. Guo H. Jiang L. Chen S. Yu C.. (2010). *DTH8* suppresses flowering in rice, influencing plant height, yield potential simultaneously. Plant Physiol. 153, 1747–1758. 10.1104/pp.110.156943 20566706 PMC2923886

[B56] Wu L. Zhang Z. Zhang H. Wang X. C. Huang R. (2008). Transcriptional modulation of ethylene response factor protein *JERF3* in the oxidative stress response enhances tolerance of tobacco seedlings to salt, drought, freezing. Plant Physiol. 148, 1953–1963. 10.1104/pp.108.126813 18945933 PMC2593663

[B57] Xu Z. S. Chen M. Li L. C. Ma Y. Z. (2008). Functions of the ERF transcription factor family in plants. Botany 86, 969–977. 10.1139/B08-041

[B58] Xu Z. S. Chen M. Li L. C. Ma Y. Z. (2011). Functions, application of the AP2/ERF transcription factor family in crop improvement. J. Integr. Plant Biol. 53, 570–585. 10.1111/j.1744-7909.2011.01062.x 21676172

[B59] Xu Z. S. Xia L. Q. Chen M. Cheng X. G. Zhang R. Y. Li L. C.. (2007). Isolation, molecular characterization of the *Triticum aestivum* L ethylene-responsive factor 1 *TaERF1* that increases multiple stress tolerance. Plant Mol. Biol. 65, 719–732. 10.1007/s11103-007-9237-9 17874224

[B60] Xu Z. S. Xiong T. F. Ni Z. Y. Chen X. P. Chen M. Li L. C. (2009). Isolation and identification of two genes encoding leucine-rich repeat (LRR) proteins differentially responsive to pathogen attack and salt stress in tobacco. Plant Sci. 176, 38–45. 10.1016/j.plantsci.2008.09.004

[B61] Yamamoto A. Kagaya Y. Toyoshima R. Kagaya M. Takeda S. Hattori T. (2009). *Arabidopsis* NF-YB subunits LEC1, LEC1-LIKE activate transcription by interacting with seed-specific ABRE-binding factors. Plant J. 58, 843–856. 10.1111/j.1365-313X.2009.03817.x 19207209

[B62] Yazawa K. Kamada H. (2007).Identification, characterization of carrot HAP factors that form a complex with the embryo-specific transcription factor C-LEC1. J. Exp. Bot. 58, 3819–3828. 10.1093/jxb/erm238 18057048

[B63] Yoshioka J. Schreiter E. R. Lee R. T. (2006). Role of thioredoxin in cell growth through interactions with signaling molecules. Antioxid. Redox Signal 8, 2143–2151. 10.1089/ars.2006.8.2143 17034356

[B64] Yoshioka Y. Suyari O. Yamada M. Ohno K. Hayashi Y. Yamaguchi M. (2007). Complex interference in the eye developmental pathway by *Drosophila* NF-YA. Genesis. 45, 21–31. 10.1002/dvg.20260 17216611

[B65] Yotsui I. Saruhashi M. Kawato T. Taji T. Hayashi T. Quatrano R. S.. (2013). ABSCISIC ACID INSENSITIVE3 regulates abscisic acid-responsive gene expression with the nuclear factor Y complex through the ACTT-core element in *Physcomitrella patens*. New Phytol. 199, 101–109. 10.1111/nph.12251 23550615

[B66] Zhang G. Liu X. Quan Z. Cheng S. Xu X. Pan S.. (2012). Genome sequence of foxtail millet *Setaria italica* provides insights into grass evolution, biofuel potential. Nat. Biotechnol. 30, 549–554. 10.1038/nbt.2195 22580950

[B67] Zhang H. Huang L. Li X. Ouyang Z. G. Yu Y. M. Li D. Y. (2013). Overexpression of a rice long-chain base kinase gene *OsLCBK1* in tobacco improves oxidative stress tolerance. Plant Biotechnol. J. 30, 9–16. 10.5511/plantbiotechnology.12.1101b

[B68] Zhang S. Wong L. Meng L. Lemaux P. G. (2002). Similarity of expression patterns of knotted1, *ZmLEC1* during somatic, zygotic embryogenesis in maize *Zea mays*. Planta 215, 191–194. 10.1007/s00425-002-0735-3 12029467

[B69] Zhao B. Ge L. Liang R. Li W. Ruan K. Lin H.. (2009). Members of *miR-169* family are induced by high salinity, transiently inhibit the NF-YA transcription factor. BMC Mol. Biol. 10, 29–39. 10.1186/1471-2199-10-29 19351418 PMC2670843

