# Supplementary material for: Foxtail Millet NF-Y Families: Genome-Wide Survey and Evolution Analyses Identified Two Functional Genes Important in Abiotic Stresses
Source: Front Plant Sci. 2015 Dec 22;6:1142. doi: 10.3389/fpls.2015.01142 (PMC4687410; doi:10.3389/fpls.2015.01142)
Supplement: Supplementary Dataset S1 — Fasta file of all foxtail millet NF-Y protein sequences. [file DataSheet1.DOC]

SiNF-YA - predicted full length protein sequences (note that in some cases more than one gene model may exist).

>SiNF-YA1

MESRPGGTNLVEPRGQGAALPSVGAAMQPWWTTSGAGLGAVSPAVVAPGSGAGISLSSSPVGGSGGAGASKGAASDESSEDSRRSGEPKDGSAGQEKNHATSQMPALVSEYLAPYSQLELNQSIASAAYQYPDPYYTGMVPPYGTQAVAHFQLPGLAHSRMPLPLEVSEEPVYVNAKQYHGILRRRQSRAKAELEKKVVKTRKPYLHESRHQHAMRRARGNGGRFLNTKKTDNGSPNGNGDPEKGDQHSEHLHVPPDLLQLRQNEA

>SiNF-YA2

MLPPHLTENGTIMIQFGHQMPDYDSPATQSTSESHQEVSGMSEGSLNEHNDQSGNHDGYSKSDENKMMSALSLGNPETAYAYPKPDRTQSFAISYPYADPYYGGAVAAYGPHAIMHPQLVGMVPSSRVPLPIEPAAEEPIYVNAKQYHAILRRRQLRAKLEAENKLVKSRKPYLHESRHQHAMKRARGTGGRFLNTKQQAEGPGGGSSDAQRNGGLFTKHEHSLPPGDRHYHPRGGA

>SiNF-YA3

MTSVVHSVSGDHRAEDQHQQQKQAEPEDQQEAPVTSSDSQPTVGTPSDYVAPYAPHDMGHAMGQYAYPNIDPYYGSLYAAYGGQPMMHPPLVGMHPTGLPLPTDAIEEPVYVNAKQYNAILRRRQSRAKAESERKLVKGRKPYLHESRHQHALKRARGAGGRFLNSKSDEKEENSDSSHKEKQNGVVPHKSGQPSTPPSPNGASSANRADSHE

>SiNF-YA4

MADHHGQPPGGGGGGAEEIKEQDRLLPIANVGRIMKQILPPNAKISKEAKETMQECVSEFISFVTGEASDKCHKEKRKTVNGDDVCWAFGALGFDDYVDPMRRYLHKYRELEGDRAAAAASSRGGGPPGPDHPSTSGGPGAGAGPGPSGGGGGHFMFGAMDRSDNNSSRPF

>SiNF-YA5

MLLREMDGDPFHTMPNYDFLSGNGYSMKQLNCSNSDRDSSSTKSEQSRQDLSAVSDGSLNGQHTPTQSGLFSPSENNDSCGKRDQGMVKSVLSFGNPEADFSPPKFDYSQPFACASYTADPYYGGVLTGYSSNSIVRPQINGATNSRVPLPIEPAAEEPIFVNAKQYHAILRRRQMRAKLEAQNKLVKGRKPYLHESRHCHAMKRVRGPGGRFLNKKELQEQQQREKAPPSLHTPTGGVGKMAFGRNLCPENSTSHSPSTSSGISSVSNGGGMMAHQEHISFSSPNFLPSMNFRKENGSEKMAVNGVRHRTPS

>SiNF-YA6

MKVNAGFIHHISVKDHPVHPMSKSNHGVLSGNDHEMKHLGHKIHDRDSSSGSGQSHQEASAVSETSLNEHTSIQSDNDEGHGKHNQFTVKPVLSMGKQGSAFSPPKLDYNASFACVPYNADAYYGGVLTGYPPHAVVHPQQNQTTNAPVVLPVEPAAEEPIYVNAKQYHAILRRRQTRAKLEAQNKLVKGRKPYLHESRHRHAMKRARGSGGRFLNTKQLQEQNQQHQASGGSSCSKVIGNTISSQSDPNPTTPSAPASSDTASASRTNQDRTCFPSVGFRPAMNFSEQGGGSAKLVR

>SiNF-YA7

MMSFRSHEGFGQVPAGAISNGGASLPWWAPAPQLLLYGEALGQGKVAPEATATAACREARFQVVPGAQALLDPPVPPAPKASAAERGSLPEVLKFSVAQGKGEKGAEYSATVALPSPFAIYNGRFELGLGQSMVSANNPYADQHYGLLSPYPVGATNGGCTRIPLNMPTEAPIYVNAKQYEGIIRRRRARAKAERENRLVKARKPYLHESRHLHALRRARGSGGRFLNTKKESNGKDAGGDGKAMISKPLMRQVASPSSEIQQSDLGNPSSVSSLSGSEVSSIYDHEDVDHYHSFDHLRTPFFTPLPSIMDGEHGGNPFKWPTASEGCCDLLRA

>SiNF-YA8

MRSAAMGFHEHGPLGFQLTTDGGRGHGGGGATAAPWWAAAQGQGCAASKFPISSGDSDPWQDLKYHEPSTAAIAAYPELHKYHAPFDLALGQSMVWSNNADAGQGQSFGLYSPYGAQPMH

GRVLLPPAIAADEPVYVNAKQFNGILRRRLARAKAARDLRVSRNRKPYLHESRHLHALRRARGTGGRFLSTSSLAAGDQAPPLASTSLGGPEPTKGSASTPARLQPGQVRQDVFLSPLIN

MAGNGDGQARWASAAPRACCDLLKV

>SiNF-YA9

MMSFKGHEGFGQVAAGGQASHGAALPWWAGPQLLYGEPAPLSPEETRREGQFQVVPGAQGTPDPAPPAAAKRGSPEVLKFSVFQGNSESDGKGEKVPEHSTTVSLQSPFPEYNGRFEIGLGQSMAPSNYSSADQCYGMLTTYGMRSMSSGRLLLPLNAPADAPIYVNPKQYEGILRRRRARAKAERENRLAKGRKPYLHESRHLHAMRRARGSGGRFLNTKKEGAANANGSGRTAAAAPPARFATSPSSEPPRAPPGLGNVSNQRCHSRSSVSSLSGSEVSSIYDHHEDHASHHRQYGGGMVRAPPFFTPLPAIMDGDHGGAAAIPSFKWAASDGCCELLKA

>SiNF-YA10

MAFRNRARGGGRRRGMVRVDGPRAGQAAEGAWHAAADQEGGGACAGHLPAAAIWLLRRRRRKHPASLVDEEEGGRRLEAASRPASMVVSSSLAIVCFACKGEKGSEHSATVALQPPFAVYNGRFGPGLGQSMVCADTTDHAKQFEGIFHWRRARAKTERVNRLVKARKPYLQESRHLHALCRARGSGGRFLNTKKIQQSDLGNLSSVSSLSGSEVSSIYDHEDVDHYHSFHHLYIPFTHSRASWTWPAASEGCCDLLRA

SiNF-YB - predicted full length protein sequences (note that in some cases more than one gene model may exist).

>SiNF-YB1

MDPMDIVGKSKEDVSLPKSTMFKIIKEMLPPDVRVARDAQDLLVECCVEFINLLSSESNEVCSREEKKTIAPEHVLKALSDLGFREYIDEVYAAYEQHKLDTLDSPKAGKFTGIEMTEEEAVAEQQRMFAEARARMNNGAPKPKEPEQEAQQQPHPQLQLHTPPQQPMQPQLQLHSPTQQSVQPQLQLHPQVQPHPQQSQVQPHPQQSQVQPQQAPQVQLHPQPQQAPQVQLHPQPQQSPQVPLQSQPQQTPQVPLHPSPEQPSQPQPQPQVHLQSQEPPQVQLQAQLQPQVQTEHGVDS

>SiNF-YB2

MADDGGSHEGGGGGGVREQDRFLPIANISRIMKKAVPANGKIAKDAKETLQECVSEFISFVTSEASDKCQKEKRKTINGDDLLWAMATLGFEDYVDPLKIYLQKYREMEGDSKLSTKAGEGSVKKDAISPHGGTSSSSNQLVQHGVYNQGMGYMQPQYHNGDT

>SiNF-YB3

MADAPASPGGGGGSHESGSPRGGGGGGGGGVREQDRFLPIANISRIMKKAIPANGKIAKDAKETVQECVSEFISFITSEASDKCQREKRKTINGDDLLWAMATLGFEDYIEPLKVYLQKYREMEGDSKLTAKAGDGSIKKDVLGHGGASSSATQGMGQQGAYNQGMGYMQPQYHNGDISN

>SiNF-YB4

MPDSDNDSGGPSNAGGELSSPREQDRFLPIANVSRIMKKALPANAKISKDAKETVQECVSEFISFITGEASDKCQREKRKTINGDDLLWAMTTLGFEDYVEPLKHYLHKFREIEGERAAASSGSAAAQQQQGDVARSAANAGGYAGYGAPGAGGMMMMMRQPMYGSPQQQQHQQPPPPPQQQQQQHQQHHMAMGGRGGGGGGSGGGGGSSSSSGLGRQDRA

>SiNF-YB5

MPDSDNESGGPSNAEFSSPREQDRFLPIANVSRIMKKALPANAKISKDAKETVQECVSEFISFITGEASDKCQREKRKTINGDDLLWAMTTLGFEDYIEPLKLYLHKFRELEGEKLATGAAGSSGSGSALQPQRETTPSAHNGAGGAVGYGMYGAGAGGGSGMIMMMGQPMYGSPPGASGYPQPPHHQMVMGAKGGSYGHGGGGSSSPSGLGRQDRL

>SiNF-YB6

MDSSSGFLPAGAANGGSNNGGGAQAQQQAAPPPIREQDRLMPIANVIRIMRRVLPPHAKISDDAKETIQECVSEYISFITGEANERCQREQRKTITAEDVLWAMSRLGFDDYVDPLSVYLHRYREFEGEARGVGGLPPGATRGGDHHHHSMAPPPMLKPRAPGAAMPPHHHDMQLHHASMYGGAVPPHHGHGHGFAMPHHQGGHHQYLPYPYDPAYGGEHAMAAYYGGSGAAYAPGNGGSGGDGSGSSGGSASQGGGFEHQHPFASYK

>SiNF-YB7

MKSRKGYGQQQGHLLSPVGSPPSDNESGAAAAAAAAWCGSSAGYCGGDSPAKEQDRFLPIANVSRIMKRSLPANAKISKEAKETVQECVSEFISFVTGEASDKCQREKRKTINGDDLLWAMTTLGFEAYVGPLKSYLNRYREAEGEKAAVLGGAGARHGDGGGGVADDGADMLGAGGGAAAAGIDRAGGHDAGGGGSADVGLMMGVSVGFGAGGGTSYYAAAAGKAYGAGDGSKVVEFDGDEENGGGMQRGFGGHLHGAVQW

>SiNF-YB8

MGRKGKRGAIREKKGGRDGEKAAPPADDDCASSSDGEGGAAAAGLPMANLVRLIRQVIPKGVKVSTRAKHLTHDCAVEFVGFVAGEAAEQAKAQHRRTIAPEDFICAFQALGFDDYVQPMSTYTRRYHEHHNNAARGYRGSFVPRPPPPPPDVAVAEEAAVTAPGVPCFSDEEMQYLRSTVPSLHGEQDDEGSSSAYSPTPAGHGYGYTGDM

>SiNF-YB9

MSMDPKGPRAGASCTMPTEPTAVAKEAAATELPPVPSTEPPVIREQDRLMPVANVSRIMRRGLPPHAKISDDAKEVIQDCVSEFISFVTGEANERCHTEHRKTVTAEDLVWALDRLGFDDYVGPLNAFLQRMREIEGGGDGGRGSSWRGPRRGSSLQVALHSAQTIRPAVYRHPAYAVGPVPRPVPGSTAAARFGGRYQMPSVGGQRSMAPYYGGAAFQAGGSRHGAFYADEASSSNEAPPAPRAGSRR

>SiNF-YB10

MADHHGQPPGGGGGGAEEIKEQDRLLPIANVGRIMKQILPPNAKISKEAKETMQECVSEFISFVTGEASDKCHKEKRKTVNGDDVCWAFGALGFDDYVDPMRRYLHKYRELEGDRAAAAASSRGGGPPGPDHPSTSGGPGAGAGPGPSGGGGGHFMFGAMDRSDNNSSRPF

>SiNF-YB11

MSEAEGAPEAGGGGSFGGKEQDRFLPIANISRIMRRGVPDNGKIAKDAKESVQECVSEFISFITSEASDKCMKEKRKTINGDDLIWSLGTLGFEEYVEPLKHYLKLYRETEGDTKGSKSSDQAGKKEILLSVEPGSSFDGL

>SiNF-YB12

MERASTSRDGNSGAVGHDNLLPIANVGRIMKEALPPQAKISKRAKETIQECATEFVGFVTGEASERCRRERRKTINGDDICHAMRSLGLDHYADAMRRYLQRYRESEELAAALNSGSGSGSGGGIQIDVRAELSIFRGHEQQDRN

>SiNF-YB13

MDNQPKNHQDGATEAPEERKIPRATVARIMRKATPPNSKIGADAKEAVDQCLVEFAAFITQVAAEECRRDKRTTVTGDDLILAFKNLGFDDYVGTLTLYLRRYREIEGNMPRARHSTMRSQGAPPAPAALTVEAAAAPSSGLTLQLGPPSVPDVTELGLHADVYAVWRGAAAPAAAGTSQAPSGADEEE

>SiNF-YB14

MDNQPKNHQDGATEAPEEHKIPRATVARIMRKATPPNSKIGADAKEAVDQCLVKFAAFVTQVAAEECRRDKRTTVTGDDLILAFKNLGFDNYVGPLTLYLRRYREIEGNMPRARHSTMRSQGAPPAPVALTVEAAAAPSSGLTLQLGPPSVPDVTELGLHADVYAVWRGAGPAPAAGTSQAPSCADEEE

>SiNF-YB15

MARKRTKEAEPQPAAAAQEEPAPAPAAPAAAMTEAEVEELPKAIVRRLVKDKLAHIAGGGEGAEVIVNKDAMAAFAESARIFIHYLSATANDMCKESKRQTINADDVLNALDEMDFPEFVEPLRTSLQEFRNKNADRRSEANKKQKEKRRKLNEEPHPQNENDPVDDAKEDDD

SiNF-YC - predicted full length protein sequences (note that in some cases more than one gene model may exist).

>SiNF-YC1

MEPSSSQPQPVTGVGSQAYPAAYVPPAMVPGAPSVVPPGSQPAAPFPNPAQLSAQHQMVYQQAQQFHQQLQQQQQQQLREFWTTQMDEIDQTTDFKNHTLPLARIKKIMKADEDVRMISAEAPVVFAKACEVFILELTLRSWMHTEENKRRTLQKNDIAAAITRTDIYDFLVDIIPRDEMKEEGLGLPRVGLPPAMGASADSYPPYYYMPAQQMPPGGGMMYGGQQGHQVTYMWQQPQGQEEEPPEEQQQQQRSA

>SiNF-YC2

MEPSSQPQPAMGVAAGGSQEYPAPAYPPAATIAAPSAVPPAGLQPGQPFPANPAQMSAQHQIVYQQAQQFHQQLQQQQQRQLQQFWAERLADIEQTTDFKNHTLPLARIKKIMKADEDVRMISAEAPVVFAKACEIFILELTLRSWMHTEENKRRTLQKNDIAAAITRTDIYDFLVDIVPRDEMKEEGVGLPRAGLPPMGAPADAYPYYYMPQQQVPGAGMVYGAQQGHPVTYLWQEPQEQQEQPPEDQQPLHGSG

>SiNF-YC3

MDEIEQTTDFKNHTLPLARIKKIMKADKDVRMISAEVPVVFAKACKVFILELTLRSWMHTEENKRRTLQKYDIAAAITRTDIYDFLVDIIPRDEMKEEGLGLPRVGLPAAMGPQLIIQGHPMTYMWQKPQGQEEEPPKSSSSSLPESSQG

>SiNF-YC4

MDNQPLPYSTGQPPATGGAPVPGVPGAAGPPPVPHHHLLQQQQAQLQAFWGYQRQEAERASASDFKNHQLPLARIKKIMKADEDVRMISAEAPVLFAKACELFILELTIRSWLHAEENKRRTLQRNDVAAAIARTDVFDFLVDIVPRDEAKEEPGSALGFAAAGPGAVGGGAAPAARMPYYYPPMGQPAPMMLAWQSASYCEEGQGFAAAGHGGAASFPPPAPPSSE

>SiNF-YC5

MDNQPLPYSTGQPPATGGAPVPGVPGAAGPPPVPHHHLLQQQQAQLQAFWGYQRQEAERASASDFKNHQLPLARIKKIMKADEDVRMISAEAPVLFAKACELFILELTIRSWLHAEENKRRTLQRNDVAAAIARTDVFDFLVDIVPREEAKEEPGSALGFAAAGPGAVGGGAAPAAGMPYYYPPMGQPAPMMPAWQVPAWDPAWQQGAAPDVDQSASYGEEGQGFAAAGHGGAASFHPPAPPSSE

>SiNF-YC6

MLGPAAQQQLQIFWNEQYREIEATTDFKNHNLPLARIKKIMKADEDVRMIAAEAPVVFARACEMFILELTHRGWAHAEENKRRTLQKSDIAAAVARTEVFDFLVDIVPRDEAKDAEAAAAAGMGAGIPHPAAGMPATDPMGYYYVQPQ

>SiNF-YC7

MDQHSQINKVVGGATNVDTPAASYALTPFLQHVPMESVPPSSVGAAFLPFSASQGSVAYPSAILPLLSNQQPSQTDPFEAATNDAAYALTALTTYNHQPEALASVPPSSSAGATFLPAPASQGAPTAYQTAIPPPLSNQHQAALLQKLQQQQLQAFWAGQLAEAEQATDLKVHSLPLARIKKIMKADEDVKMIAAEAPVVFAKACEMFILELTLRSWLHTEGTKRRTMQRSDVSAAIMAEMFDFLMDVTPTEQQTNGDGVLPPPPPLQTTAGQVPFPMHVPFPMYANHQPPFMWPTPEYQQQQNPGGGNE

>SiNF-YC8

MATAAAAAVDEPTVAMEEELRKEGELVEEPTEAMEQVAEDREEIGGPAEATEQVEEEREEATDPAEPMEQVGEDREEAEAEEGASLRPALPVGRVKRIMRVDRDIKKVTSEATLLIAAATELFLGSLAAGAHTAAARRGRRAVRAAHVRAAARAHRPTADFLLDCLHAEEEAPRARPVAGSAGGGGGREAKPLPRGTRRIDAFFQKVT

>SiNF-YC9

MRKKLDTRFPAPRIKKIMQADEDVGKIALAVPVLVSKALELFLQDLCDRTYDITIRKGVKTVGSSHLKQCIQTYNVYDFLREVVSKVPDTGTSDAIADDKLGKRRKAEEDGSEEELKRTRNEAESHTSNGRGRGRGRGRGRRGGRGAWKEVVITHEQFVENQSSKPAGLKVEIADEVPDATEAKEATPVSSARASIRNIDLNLDPAEEDDEVAVPPEAQPSAPATDPAAANLGLTAPATSSAAANLGLTTPATSSAATTAGPSVPGLNEGAKLKDLLGGWELPDMNKMDMDPVQFALSSNHKLDDDEDYDNED

>SiNF-YC10

MRKKLGTRFPAARIKKIMQADEDVGKIALAVPVLVSRALELFLQDLIDRTYEITLQSGAKTLNSFHLKQCVKRYSSFDFLTEVVNKVPDLGGADSCGDERGLPRRRKSNGSDPENDESRSSKMAIRSVNISPRGRGRGRGRGRGRPPTKRKEVGYVQFEDESSMFAEQGEPLPGEETVPESNHNNENIPPSAQPPQEAPLPAAVPGTSSKVEEANTDHQSDWPMPDAAIGSIGVGPSGFGHLTVQVDEDEDYDNED

>SiNF-YC11

MSNGSKERFGHPQELHGLPPTPLTSLQQKELDEFWRKTVEDIENTMNFDNHILPMSYVAKIIRDNQGSLMISSETPSCLTKVLEIFIQELTLRAWMCAKSHDRSSTILESDIYEAINSKESYVFLNDVLQRLETNHTQASMSSNAPQLHQESHFLAATSTLKENGAMDPLTKPRDQAFQIPKDNLVPAINAQPDPLELKNDEDLTMSTTSSGSIEEAK

>SiNF-YC12

MDHRIEIRSSPRTPMPLSQQEMIDDFWRKRQIEIEAIQDFGDRAIPMTRIKKVICDEKGKMMMTFDTPSFLTKACEIFVQEIAFRAWMCANSNQRSIILDSDITEAIASTQSYDFLNDFLNAHQDEHHSCPYPKPTKKRHDRLLTNLPSSSCHLPPHQNQLPQFMPQTNGPRVPLSLASLPHEASSVIATTGTPAPIVSGIIPPTNYMANGLGSFGNTINNIAASSGVMNHLKELPRALANIPNTYCYMNMVASASVYGVDSASSSNVAAQDSGIAFHCPYIPQITLQLPSPLQTTISSAHTTTNITIENYIHVGVAATKSTIHASSKTNGNGDIDPNAIGVGDDQHQHEEEANTSLEVNGVHGSLNAQAVAATSIGNDINWDEFDMLDDSLLSVVGKDIVMDEEPGPLPNTASNDDLLLASNMSDLEGFSHEPYLLDDIISSAGTSKRCT

>SiNF-YC13

MDHAIDIHASSKKPALESKQQMIREFWRKKQEEIEAIEDFSKHAIPMRRLKKVISANKGKIMMRFDTPSFLTKVCEIFVQELSFRAWMCAHSQDRGVILDSDIADAVASIEPYDFFNNVLPTDLEEYNSSLRSKPIKKHHHLLIDKPSTPTHLPSDQYQMPQFIPQSVGHSTCVHISPPLSPKTGCRVPLSLTCVPQEPYPLMPTTITPAPIVSGRMVFLRNNISNNVDILGVTTPLPVPPSAQPNIPNNRYISTIASTSSDCVGYANTSNVVTQDGGSSALQCSSPSPIANNSGPIATCLNHMKPEVAQIKNDIHAHGTDGIDPEATTGVNDGQNQHGSLDAEVSTIANVSGYSSNINWDEVDMADDSLLIEFWEDIFMNKDPAPSPTATSTTDHVPFPCDMPKLEGFGHELYLLDDIVSSASTSRRLS

>SiNF-YC14

MRQARPYSGIFCGGVSARTGPHALPLARIKKIMKRSAGEAADGGARMISGEAPVVFSKACELFIAEITRRAWAATLEGKRRTVHKEDVATAVHNTDLFDFLVDVVMADAGGGGHAAPGYDDDENGALE
