# Supplementary material for: Foxtail Millet NF-Y Families: Genome-Wide Survey and Evolution Analyses Identified Two Functional Genes Important in Abiotic Stresses
Source: Front Plant Sci. 2015 Dec 22;6:1142. doi: 10.3389/fpls.2015.01142 (PMC4687410; doi:10.3389/fpls.2015.01142)
Supplement: Supplementary Dataset S2 — Fasta file of all foxtail millet NF-Y protein conserved regions. [file DataSheet2.DOC]

These are the Stetaria italic NF-Y, conserved regions only.

>SiNF-YA1

EEPVYVNAKQYHGILRRRQSRAKAELEKKVVKTRKPYLHESRHQHAMRRARGNGGRFLNTK

>SiNF-YA2

AEEPIYVNAKQYHAILRRRQLRAKLEAENKLVKSRKPYLHESRHQHAMKRARGTGGRFLNTK

>SiNF-YA3

EEPVYVNAKQYNAILRRRQSRAKAESERKLVKGRKPYLHESRHQHALKRARGAGGRFLNSK

>SiNF-YA4

ENEPIYVNPKQYHGILRRRQLRAKLEAQNKLVRARKPYLHESRHLHAMKRARGSGGRFLNTK

>SiNF-YA5

AEEPIFVNAKQYHAILRRRQMRAKLEAQNKLVKGRKPYLHESRHCHAMKRVRGPGGRFLNKK

>SiNF-YA6

AEEPIYVNAKQYHAILRRRQTRAKLEAQNKLVKGRKPYLHESRHRHAMKRARGSGGRFLNTK

>SiNF-YA7

TEAPIYVNAKQYEGIIRRRRARAKAERENRLVKARKPYLHESRHLHALRRARGSGGRFLNTK

>SiNF-YA8

VYVNAKQFNGILRRRLARAKAARDLRVSRNRKPYLHESRHLHALRRARGTGGRFLSTSS

>SiNF-YA9

ADAPIYVNPKQYEGILRRRRARAKAERENRLAKGRKPYLHESRHLHAMRRARGSGGRFLNTK

>SiNF-YA10

DHAKQFEGIFHWRRARAKTERVNRLVKARKPYLQESRHLHALCRARGSGGRFLNTK

>SiNF-YB1

KEDVSLPKSTMFKIIKEMLPPDVRVARDAQDLLVECCVEFINLLSSESNEVCSREEKKTIAPEHVLKALSDLGFREYIDEVYAAYEQHKLDTLD

>SiNF-YB2

REQDRFLPIANISRIMKKAVPANGKIAKDAKETLQECVSEFISFVTSEASDKCQKEKRKTINGDDLLWAMATLGFEDYVDPLKIYLQKYREMEGD

>SiNF-YB3

REQDRFLPIANISRIMKKAIPANGKIAKDAKETVQECVSEFISFITSEASDKCQREKRKTINGDDLLWAMATLGFEDYIEPLKVYLQKYREMEGD

>SiNF-YB4

REQDRFLPIANVSRIMKKALPANAKISKDAKETVQECVSEFISFITGEASDKCQREKRKTINGDDLLWAMTTLGFEDYVEPLKHYLHKFREIEGE

>SiNF-YB5

REQDRFLPIANVSRIMKKALPANAKISKDAKETVQECVSEFISFITGEASDKCQREKRKTINGDDLLWAMTTLGFEDYIEPLKLYLHKFRELEGE

>SiNF-YB6

REQDRLMPIANVIRIMRRVLPPHAKISDDAKETIQECVSEYISFITGEANERCQREQRKTITAEDVLWAMSRLGFDDYVDPLSVYLHRYREFEGE

>SiNF-YB7

KEQDRFLPIANVSRIMKRSLPANAKISKEAKETVQECVSEFISFVTGEASDKCQREKRKTINGDDLLWAMTTLGFEAYVGPLKSYLNRYREAEGE

>SiNF-YB8

GAAAAGLPMANLVRLIRQVIPKGVKVSTRAKHLTHDCAVEFVGFVAGEAAEQAKAQHRRTIAPEDFICAFQALGFDDYVQPMSTYTRRYHEHHNN

>SiNF-YB9

REQDRLMPVANVSRIMRRGLPPHAKISDDAKEVIQDCVSEFISFVTGEANERCHTEHRKTVTAEDLVWALDRLGFDDYVGPLNAFLQRMREIEGG

>SiNF-YB10

KEQDRLLPIANVGRIMKQILPPNAKISKEAKETMQECVSEFISFVTGEASDKCHKEKRKTVNGDDVCWAFGALGFDDYVDPMRRYLHKYRELEGD

>SiNF-YB11

KEQDRFLPIANISRIMRRGVPDNGKIAKDAKESVQECVSEFISFITSEASDKCMKEKRKTINGDDLIWSLGTLGFEEYVEPLKHYLKLYREGD

>SiNF-YB12

HDNLLPIANVGRIMKEALPPQAKISKRAKETIQECATEFVGFVTGEASERCRRERRKTINGDDICHAMRSLGLDHYADAMRRYLQRYRESEE

>SiNF-YB13

APEERKIPRATVARIMRKATPPNSKGADAKEAVDQCLVEFAAFITQVAAEECRRDKRTTVTGDDLILAFKNLGFDDYVGTLTLYLRRYREIEGN

>SiNF-YB14

APEEHKIPRATVARIMRKATPPNSKIGADAKEAVDQCLVKFAAFVTQVAAEECRRDKRTTVTGDDLILAFKNLGFDNYVGPLTLYLRRYREIEGN

>SiNF-YB15

EAEVEELPKAIVRRLVKDKLAHIAGGGEGAEVIVNKDAMAAFAESARIFIHYLSATANDMCKESKRQTINADDVLNALDEMDFPEFVEPLRTSLQEFRNKNAD

>SiNF-YC1

TDFKNHTLPLARIKKIMKADEDVRMISAEAPVVFAKACEVFILELTLRSWMHTEENKRRTLQKNDIAAAITRTDIYDFLVDIIPRDEMKEEG

>SiNF-YC2

TDFKNHTLPLARIKKIMKADEDVRMISAEAPVVFAKACEIFILELTLRSWMHTEENKRRTLQKNDIAAAITRTDIYDFLVDIVPRDEMKEEG

>SiNF-YC3

TDFKNHTLPLARIKKIMKADKDVRMISAEVPVVFAKACKVFILELTLRSWMHTEENKRRTLQKYDIAAAITRTDIYDFLVDIIPRDEMKEEG

>SiNF-YC4

ASDFKNHQLPLARIKKIMKADEDVRMISAEAPVLFAKACELFILELTIRSWLHAEENKRRTLQRNDVAAAIARTDVFDFLVDIVPRDEAKEEP

>SiNF-YC5

ASDFKNHQLPLARIKKIMKADEDVRMISAEAPVLFAKACELFILELTIRSWLHAEENKRRTLQRNDVAAAIARTDVFDFLVDIVPREEAKEEP

>SiNF-YC6

TDFKNHNLPLARIKKIMKADEDVRMIAAEAPVVFARACEMFILELTHRGWAHAEENKRRTLQKSDIAAAVARTEVFDFLVDIVPRDEAKDAE

>SiNF-YC7

TDLKVHSLPLARIKKIMKADEDVKMIAAEAPVVFAKACEMFILELTLRSWLHTEGTKRRTMQRSDVSAAIMANEMFDFLMDVTPTEQQTNGD

>SiNF-YC8

ASLRPALPVGRVKRIMRVDRDIKKVTSEATLLIAAATELFLGSLAAGAHTAAARRGRRAVRAAHVRAAARAHRPTADFLLDCLHAEEEAPRA

>SiNF-YC9

FPAPRIKKIMQADEDVGKIALAVPVLVSKALELFLQDLCDRTYDITIRKGVK-TVGSSHLKQCIQTYNVYDFLREVVSKVPDTGTS

>SiNF-YC10

FPAARIKKIMQADEDVGKIALAVPVLVSRALELFLQDLIDRTYEITLQSGAK-TLNSFHLKQCVKRYSSFDFLTEVVNKVPDLGGA

>SiNF-YC11

MNFDNHILPMSYVAKIIRDNQGSLMISSETPSCLTKVLEIFIQELTLRAWMCAKSHDRSSTILESDIYEAINSKESYVFLNDVLQRLETNHTQ

>SiNF-YC12

QDFGDRAIPMTRIKKVICDEKGKMMMTFDTPSFLTKACEIFVQEIAFRAWMCANSNQRSIILDSDITEAIASTQSYDFLNDFLNAHQDEHHS

>SiNF-YC13

EDFSKHAIPMRRLKKVISANKGKIMMRFDTPSFLTKVCEIFVQELSFRAWMCAHSQDRGVILDSDIADAVASIEPYDFFNNVLPTDLEEYNS

>SiNF-YC14

HALPLARIKKIMKRSAGEAADGGARMISGEAPVVFSKACELFIAEITRRAWAATLEGKRRTVHKEDVATAVHNTDLFDFLVDVVMADAGGGGH
