# Supplementary material for: Foxtail Millet NF-Y Families: Genome-Wide Survey and Evolution Analyses Identified Two Functional Genes Important in Abiotic Stresses
Source: Front Plant Sci. 2015 Dec 22;6:1142. doi: 10.3389/fpls.2015.01142 (PMC4687410; doi:10.3389/fpls.2015.01142)
Supplement: Supplementary Dataset S3 — CDS and protein sequences of SiNF-YA1 and SiNF-YB8. [file DataSheet3.DOC]

SiNF-YA1 full length CDS sequence.

ATGGAGTCGCGGCCGGGCGGAACGAACCTGGTGGAGCCCAGAGGGCAGGGCGCCGCGCTGCCGTCGGTCGGCGCGGCGATGCAGCCGTGGTGGACGACCTCCGGGGCCGGGCTCGGTGCGGTGTCGCCGGCCGTTGTGGCGCCAGGGAGCGGGGCAGGGATTAGCCTGTCGAGCAGCCCAGTAGGTGGTAGTGGTGGTGCCGGGGCGTCCAAAGGCGCCGCGAGTGACGAGAGCAGCGAGGATTCACGGAGATCTGGGGAACCAAAAGATGGAAGTGCTGGTCAAGAAAAGAACCATGCCACATCACAGATGCCTGCTTTGGTGTCAGAGTATTTGGCACCATATTCACAGCTGGAACTGAACCAATCAATTGCTTCGGCAGCATATCAGTACCCAGATCCTTACTACACTGGCATGGTTCCTCCCTATGGCACTCAAGCTGTGGCTCATTTCCAGCTACCTGGATTAGCCCACTCTCGCATGCCATTACCCCTTGAAGTATCGGAGGAGCCTGTTTATGTAAATGCAAAGCAGTACCATGGAATTTTAAGGCGAAGGCAGTCACGTGCCAAGGCTGAACTTGAGAAAAAGGTGGTCAAAACCAGGAAGCCCTACCTTCATGAGTCTCGTCATCAACACGCAATGAGAAGGGCGAGAGGAAACGGGGGACGCTTCCTGAACACAAAGAAAACTGACAATGGTTCTCCCAACGGTAATGGTGATCCTGAGAAAGGAGACCAACACTCAGAGCATCTCCATGTCCCTCCTGACTTACTACAGCTACGACAGAACGAGGCATGA

SiNF-YA1 full length protein sequence.

MESRPGGTNLVEPRGQGAALPSVGAAMQPWWTTSGAGLGAVSPAVVAPGSGAGISLSSSPVGGSGGAGASKGAASDESSEDSRRSGEPKDGSAGQEKNHATSQMPALVSEYLAPYSQLELNQSIASAAYQYPDPYYTGMVPPYGTQAVAHFQLPGLAHSRMPLPLEVSEEPVYVNAKQYHGILRRRQSRAKAELEKKVVKTRKPYLHESRHQHAMRRARGNGGRFLNTKKTDNGSPNGNGDPEKGDQHSEHLHVPPDLLQLRQNEA

SiNF-YB8 full length CDS sequence.

ATGGGTCGCAAGGGAAAGCGTGGTGCGATTCGTGAGAAGAAGGGCGGCCGTGACGGCGAGAAGGCCGCGCCGCCGGCTGACGACGACTGCGCGTCGTCGTCGGACGGGGAGGGAGGCGCCGCGGCCGCCGGGCTGCCGATGGCCAACCTCGTGCGCCTCATCCGGCAGGTGATCCCCAAGGGCGTCAAGGTCTCGACGCGAGCCAAGCACCTCACCCACGACTGCGCCGTCGAGTTCGTCGGCTTCGTCGCCGGCGAGGCGGCCGAGCAGGCCAAGGCGCAGCACCGCCGCACCATCGCGCCCGAGGACTTCATCTGCGCGTTCCAGGCGCTCGGGTTCGACGACTACGTCCAGCCCATGAGCACCTACACCCGCCGCTACCACGAGCACCATAATAACGCCGCCCGCGGCTACAGGGGGTCGTTCGTGCCACGCCCTCCTCCTCCACCCCCTGATGTGGCGGTGGCGGAGGAGGCGGCCGTCACGGCCCCTGGCGTGCCATGCTTCTCCGATGAGGAGATGCAGTACCTGAGGTCGACGGTGCCTTCCCTGCATGGAGAACAAGATGACGAGGGAAGCTCCTCGGCGTACTCGCCGACGCCGGCCGGGCACGGCTACGGTTACACCGGAGACATGTGA

SiNF-YB8 full length protein sequence.

MGRKGKRGAIREKKGGRDGEKAAPPADDDCASSSDGEGGAAAAGLPMANLVRLIRQVIPKGVKVSTRAKHLTHDCAVEFVGFVAGEAAEQAKAQHRRTIAPEDFICAFQALGFDDYVQPMSTYTRRYHEHHNNAARGYRGSFVPRPPPPPPDVAVAEEAAVTAPGVPCFSDEEMQYLRSTVPSLHGEQDDEGSSSAYSPTPAGHGYGYTGDM
