# Supplementary material for: Foxtail Millet NF-Y Families: Genome-Wide Survey and Evolution Analyses Identified Two Functional Genes Important in Abiotic Stresses
Source: Front Plant Sci. 2015 Dec 22;6:1142. doi: 10.3389/fpls.2015.01142 (PMC4687410; doi:10.3389/fpls.2015.01142)
Supplement: Supplementary Dataset S4 — Fasta file of tobacco ERD10, CAT, SOD, POD, and LEA5 promoters. [file DataSheet4.DOC]

Fasta file of tobacco ERD10C, CAT, SOD, POD and LEA5 promoters.

>ERD10C promoter

CTATCATTTCGCCACGTATCACGTTGTTGTACCCCTCACCAATACCGACACCCTTGTTCC

AAAAGGATAACCAAACGAGGCCGACACTATCTGGT

CCCACAATTACAACCTTACACGTTGGCATCCTACGTGTATTGATATTATTGGTATCCTTC

GCCGTCACAGTTCAGCTCCACATTTCTACTTTATTCTTACTACTCCCTTTGTCCGAAAAT

AAGAATAGTAGAGAAAAAAGATGTATTAATTAAATTAACCTTAATTAATTATTAACTTGA

TATATTAGAATATATAAATAAGGATATATTTTTAAAAAATAAAATTGCTTTTCTTCTTGG

TTATCTAAATGAATATTTATTTTGAATCAAAATAAAAAGATAAAATGGTCACTTAACGTG

GATCAGAGAGAGTAATTCTTTAATTCAAAGTATTCAACTCCGTTGTTGTGGAGTAAGCAAACTGCTCTCCTTATTCCGTGATAATAGACTACATAAGAATACAAAGATGAATATGTGTCAAATGAGCAGTTTGGTACCTTATTTCGATTGATTAAAACCAATTGATATCAATAATATTAG

TTGGAACATAATTTGCTCGATATTTGCTTGATTTCAAAGGGATAAGCTAATTTGAGCCTA

ACAATTGGCTATAATATGGATTTTATTTAATAGATGTTATTACGCCTTCAACCTCAAAAA

GATAGAATCCTACTCAACGAAATATGTAGGTATTTATGGCAGAACGAGTCAATCTTTTTT

TTTACAATCAAACAATAGACGAAACTGTTATGAGTATACTTTATATAAAGTAAATGATCT

AAGCTTCACTGCTTCGATTTATTATTCGCATCAAATCTTAGGTTCTCTATTGCACTCCCT

AAATAGAGTTTTTTTTCATCTGTTCCTCGTGACATTAGTACGTTATTGTTCATTGAGAAA

TACTTAAGCTTAGAAGGTATATAGTCCCCTTAAGTCATTCGCACAACTCAACATCATTTT

AATTTATTTGTTTCTTATTTGAAAACTAAATTGCTAAATCATACTAACAAAACTTTTTCT

AATTTATAATAACTATATAAAATTAATTAAATTAAAAATTTACGAGTGAATTTCAGGACA

TATGCAACTCCTATTGTTCCAAACTTATCGAGTGGTTAATTAACTTTCGAATCTGGACAG

AGTTTACGGACTATATATTTTTCACCAGCTAAATTTGAATACCCACGAAATCCAGTAATA

TTTTGCCACGTCACTATAATAAGGTGAATATAAATTAATATACTCTCGGAGTTGCGAAAC

AGTACATAGGAGACAGAACAAAAGAGGCCACGTCGACGTCTGTTGCACTTGGTGTACTTGATTGGTATTTACTGAATATTATGAGTCCTTTGGACTACTTTTCTTATTATTTATAATAAA

ATTATCTTATCTCAATCGCGTAACGTCCGCGTTATGATTGCTATAAAAGGCGCACTAAGT

CTCTAGTTTCTAACACTTCCAATTCAATATATTCAAACTTTTACTTTAGCTCAAAGTGTC

ATCATTCTTTGCTTTGTTCTTAGCAACAAGTTTTTCTTTGTTTTTTGATTGAAAAAAAAA

TTTTTTTTTCAATCAAAAAACAAAGAAAAACTTGTTGCTAAGAACAAAGCAAAGAATGATGACACTTTGAGCTAAAGTAAAAGTTTGAATATATTGA

>LEA5 promoter

GTTTTGAATGGTTGTTATTAGAATAGATTGGGCATAACTTAAGGAGCTTGAATCTCAGTTTTGGAATGATTTTGTGAAATTTTGAAGTGGTTTGAATTGAAAATTCGAAGTAAAAGTTGAATAGAAAAAAATATGATATGTGTATCACACTGTGTATCATTTATGTATCACATATGTATCATATTTGTATCAATTATGTATCATATGTATATCCATGTATACCTGTGTGCGCGATACATGCGTGATACATGTGTCGCAGAAGACTTTTTTGAACTCGATTTAATTATGAATTTTGATCAAAACCAGTCCAAATCACCTCCAATCTTCCTCAAATTTTGTATATTGACTTATCCATATATTTTCAATAAATTCAACTATACCCATTGAAAAAGTTCCTTTTTTGTTTAGATTTTTGGAATTTGTATAGATTTTTGTATTTCATCACCTTATTTGCTACCCCGTTCATGAAATTTCCTTTCCGTCTTGTATTTAGTATTGCTAATCACGCTTAAAAATATGGAAGATAATTTTGCATGTGATTCTTTGATAGAAAGAACCCTATTATTTGGTTTTTCAGTTGGCTAGTTTTAATAAATCTATGACTAATGGCTAGAGATTAGTAAGTTGAGATTTATTTGGGACATTTGTGTAAGTTTCCCTTTATATTTTATACTAATAGCCTATTTTTATGTAGCTTTAATTTTATAAAATAATTCTAAAAAATTAAATTTGGAGTTGAGATCAAAATTTAAAAAGTTTGGACATCTTACTCCTAATTTCATCGGGAGTACTACTCCTTTTCTTTTTGAAACAAAACAAAAAAATTAATTAAACAAGGCAAAAAGAGAAGATCACGAGACAACTTCAAGAATCTGTAAAATTAAGGAAAAGTCCCCGCGTCAACTGGTCAGCTTTTATAGGACCTCTGACGCGTGGTTGCAGATTCACGTGTCCACTTATGCTGATTGATTCAACTGCAATGCAAAGTCCTTCACTCAATCTCTTGCGCCGTCTCCAAGCCTTAGATTTTTTTCTGACTACATATTTATTTTTAGAAATATTTTTTTATTATAATATCAGAGTAGGTAAAAGTCTTGCATTAGAGTCTCACCATTATTCAATATAAAAAAGAATTTTTACGTGCTTAACTCATAAATTAGTATCAAGCCCGCACTTGAGGGCGCGTATTAAAGATATATTAAATAAATAAAAATAAAAATATACTTTTTGTAACAATTTAAACTTTTAGATTAGATGGTCACGCAATATTTTATTAAAATATATTTAGACTCATATCTTAAAGTTTAATTTAAACAAAAGAAAGATATCGAACATAACTGATTCTAGTTAACATGATACACAGACATGATTAATTGATTCTTGGATTGAAAGAAAATGTAATTTCAGATGTTTACTTTTTAGTATATTTAAAAAAACATTTTAATTTTTACCCGATAAGACGTAAACTTCCTGTACACTTATCGTCGAGCAAATAAAGGATCTATATTGAATTTGAGGTGACTTTTATAATTAGGAGTATTATTGTTGTGGCCTTAATTGTCTCCCGCTGATATTTATTATACGTGAAATTGGCTCTTAGTGGCATCGACCAGGTGTCATTCAATGAGAGGACCTTTGCGTCTGAATAGAGGCCGACCTATCACGTGGACCCACTTATACACCCCCCCCCCCCCAAGTGGGTGCTCTGTTTTCCTTCGTCTCAGTCCTCCTCCTCCATGTCTATATATACATCTCACTTCCCCTTCCTATGTATCTGCTCCCAACTATTGTAGTTGAAGCGAAGTGTTTCAGTTCGCCGCCGTGGAAGTTTTATAAATAAATAGCTTCCGCCGCAGCGTTCCCTTGAAATTCTTTCGCTTCAACTCAAGCAAA

>SOD promoter

TATTTAAGATATGAAGGGAGATGATCTCTAGACAAATGTAAAAAAATAAGTACATGCAATTTTCCCAATATTCAAAAAAAATAAATATAAAAGCATTAGTATAGTTGAGCTTCTAGTTTCAAGAACTCAAAAAAGAAATTTATACGCAACCAGTTTAGAAAAATGATAAATCTTTTAGATTCAACAAGAAAAATAACTTTCATATTGCAAGTCGAACATATTTGAAACAAATATTGTATGATACGCAGAATGAAGCCATAATGTTGCTATAGTATACTATATCTAATATTACTTGACACCTCAAAGAATAATAGGTTTTCATGTGCTTGTTTAAGAGCATTTAAATTGGGATAATTAATTAATTTGTCCAAACAAATCGAGTCTAATTGCCAAGTCGACGGTATCAGACATTACGTAAATCTCTTGGTTTATTATCATGAGTGACCCACTTGGACAAAAAGAGAGTTAAAACATGGTGCTCCCACTTTAAACTGAGAATGTACTTAGCCCCTGGATTTTCACCTTCTTTTTCATACTAGTCCTTCCTAGTTTCCATTTGTCTTCTCCCTCCCAAAAGTTATTTTTCCTTCTTTTTTTTTGGGGTAAAAATATCCTACTAATTACTTTGTTATTTAGCGAAATTAAACCAGAAAATATATATTTAATAAGATAGAGAAATCAAGATGACAGGTGTGGTGAGCTCAATGAGTTAATCGGACCTCTCCGGGCATTACCTGTAATTAGGGGTGTACAAAAAAAACCGACAAATTGCACCAACCCGATAATCCGAGAAAAAAAACCCGACTATGGTTTGGTTTGATTTGGTTTGGTGTTGGAAAAAAAACCTGACCATAATTGGTTTGGTTTGGTTTTAACTAAAAAAAGTCAAACCAAAACCAAACCAACCCGACTATTTAAAGAAAGACAAACTAAAACCAAACCAACCCGATATTATATATATATATAGAAGTTATAAATATATTTATTGTGATGTAATTTATAAATATTTCTTAAACTTTTTCATAATTTTATCTTTTAAGGTATTATTTTAAGGTTGGATTTAAAACTTTTGAATGTTCTAATCCCTAAGTTTAATAGCCATTAATATTAGTAACTTAAATAATGCTAACAAAAGCCCAAACCAAAATCAAATCAATACTAATGCTAACAAAAGACATTCAATTCAATAATACGAACGACGATGTATTGAATATCTATTTTTTGTTTTGCAATAATTTAGATAAAAATGCATAATTTATTTTTATTTTTTCTTTAGCGTTTAGTCATGTAATTAATATTCTTTTATTAGTCTACTTATTTTACCATGACTTAGTACTTTTAGATTATGTTTATTTTTATTATGGCTTTCAAATTACAATATTTATATTACATAATTTTATTGTCTTTATTGTTGAATATTTTAGGATAATGTCATGACACATCTCATATTTTGTATTATTTTCTTGAAAAATACCTTATATAGTTGTATGTTACTAGGATTAAAGAAATATTTGAGCATAAGTTATATGTTTTGTGCTACGAAGATTTTACCAAAAAAACCCCGAAAAACCCGAAAACTCGAGAAAAGTCGAGATTGAAAAACCCAAATTTTATTGATTTGGTTTGATCTTTAGATTTAATAAGCAATTGATTTGGTTTGGTAATTGCAAAATACGAACCAAAGTACACCCCTACCTGTAACTATAAAAGGACACCGTAGAGTTAACAGGCAGAAAGCATTTAGGAATATCTCAAAA

>POD promoter

GAATATACTCCTACATGCACTTGATGTTTGCATCAAACCAATAGAAAGATAATATGTGTT

GCACATAGAGTTTTATTACCTAATCATAATCAATTAATGTAATTTTTATTTCATTAAATCACTAATCAAGATAAATTTAATCGATTTAGTGTAAACACCGAGTGCAAATAATTATTTATCCTTTTAAACCCTTTTTCTATCCTTCCAAATTGAAGTTTTACCATACAATTTTTGTCCATTCTAAAAAAAAGTAGAATTTTAGTTATATAAAAGCTTAAATTTTAAACTTTTTTTATAACCTTAGTTTTACAATTTTAGATAAATAACACATATTTTTCAATAGAAACTCTGAATATTTAGAAAATATTAAGGTGAAGATGGAATCATGCACCTAAATGGAGTTCAATTTATGTGAGTATTTCATTTTTTTTAGCTAATATACCACTGCCCAACGTAAGTTTATCAATAAAAAACAAAAATATATTTCTTAATATATGGCAAAAAATACGAGCTCACAAAAATAATACAATCTGAAACTAAAAAGACAATCTTTTCTTCTTCTTTTTTTATGGGTGTTATAGTCATTTCATAAATATTCAAAGTCCCTTGATATGATGGAGTCCACGTCCACGATGCACAAAAATCCAACACCCATAACAAAGAATCCTTCGTCTGACTTTCCTCCGATTTTCAATTCACTCACCTACCATACAACGAATCCATACTTTACTTCACCCTTATAAAAAACCAAATCTCAACCGTTCAATTCATATCTCAATCTAAGCCGCCCGATCATCCTTAATCCTACGGTCCACATTCACCTACTAGAACGTTCTCTTCCTTCAATTCAAAACCCTTTATATCCCCCATTTCCAGTGCTTGTGGTCTCCTCGTGCCATATTCTCTTTATAGGGTTTAACGTTTTGCCTTTTCTCCTCGACTCGATTATATCAGTTTCTTCAGGTTCTTATATTTCATTTATCTTCAGATCTCTAATGCTACTGTATTTGAGTGTTTTTTCTCTAATGCTACTGTAGTTGAGTTTTTTTTTAATTTTATTTATTTTATTCACTTTTTTTATTCTCGATTATAGTTTTCAAGCTCTTTTATGGCCTTAATGTTGTGAAATATGTGTGTAGATCCTTTAAAAATGAAATGTGTATAGATGTTTAACTACGTTATTATTTTGATCTGTGGACTATTGTTTTTTCTTGAAGTATGTGTTACTTACAGAGTCGGATCAATAGATGAAGTTGATCTCCAGCACAGCAATGTTTTAATTATTATTATTAATTTTATTTAAACTGTATGTCGATTAAAGTTTTCAAGCTCTTTTCGGTTAAATGGTGTGAAAAATGTGTATGTATGTATTTTTTTAAAATTAAAATTTGTATAGATTTTTAATCTAAAGTTTTTAGTTTGGTATGAGGTTTTAGTTTCTGAAACACCTTTTTATGGATTATGTGCTCCTAATATTAGCAGATCCATGATTTTAAAGACATTATATCAATTTATATTATTTTTAGTTTTTCAAAAACTGTATAGATTTTTTATTGCTAATGATGTGTATAAGTGTTTTAACTATGATTTAGTTTGACCAAAGGTTTTAACTAAGATATAACACACATTATATTTGTG

TTTTTTTGCTTATTTTAACTACAATGTTTTTTTTGACATATACATGCGCCTTCTGGATCA

AATCTAACAATAGATATGTATGGTAACTGTTAACGGCATATGATCTGTTAATACTTTTAT

GTGTATTGGATTCTGAAACTTGGATCTTATTGTGATTGAATGTTGTGTAGAACAATTGCT

>CAT promoter

AGCCCAATTTTGCAACCCGTCCATGAAGTGGAACAACAAGTCATCATTGGTCAGGTTGGGGATTTGAAGCATAAGGGTAGTGAACTCCTTGACATAGTTACGTATGCTCCCTGTTTGCTTCAATTCCCTAAGCTTGCGCCTTGCCTCGTACAAGACATTGTTTGGAAAGAATTGTCGCTTGAACTCCGCTTTGAACTGATCCCATGTGCTAATAGTACCTAGACCTTTATCCATGTTGTCCATCTTCCTTCTCCACCATAGCATGGCAGTCTCTAAGAGGTACAATACCGTAGTATTGATCTTGGCCTCGTCGTCCCTCACTTTGTCGTGCCTGAAGTAGTTCTCCAAGTGCCAAAGGAAGTTTTCTACTTCTTGTGCATCACGAACACCTTTGAACACCGGGGGTTTAGGAGCCTCGATTTTGGCCTCCCTCGTCACCACAACATTGCTGGCTACCTCGGTCATGCCAGCATTGACATGCTCCTCGAGTGACTCTATCTTTGCCTTCATAGCATCGATAGTACTCAAAGCCTCCATGAGTCTGCACTCTAAGGCAGTGATGGTTTGCCTTAGTTCCATCTCAGTCTGTGTACGTCCCTCCAAGTCATTTCGGATACTCTCAATCTCTTCAAGAGTGTGCCCCTCAAGAACATTAAGGGTGCCTTCCACCTTCCCCAAGCGTTGGCCAAATATCTCCACGGCATCCATCCCCGCGTTCATCTTCATCACCCACTCTTTACCAAGCGAGACGTCCTCTGGAAGGACCTCCACTTCATCCTCGCTCGCCTCAGTGGCAGATGGTTCTTGGGATGTAAGCCCTTCGTTTGACACAACCTCATGTGGCACCTCCTGGCTCTTGTTGGTGGCATTCCTCTATTTGCTACGACCGCTCTTGCCAGCAGCATCCTGGATGACGTTGGCTTGGGTGTTGGCAACGTTAATTTCTCCGCCATTCGCCATTCCCTTAGTTGAAACCTTCGCTCTGATACCACGTTGTCACGTCCTTAGTTGTTAACTAAGTACACGTGCGGCACTTGACAACTCGCTCACGATCTTGCACAAATTGCTCACGTTCTTACTATGCCAAGTCAGCCTTACTACCTTTAAGACCGCTAAGAGAATGGTAGAAAGAACACAAGAGAATTGTTCAAGGAAGCTTTGTATTAGAGAGAACTTGAATTGTTTGCTTGATGAATTACAAATGAATGACCCCCTTTATATACTAGTCTCCTAGGGGCTAGTGTGTAAATATTAATTATTACACAAGTCCTTGATATTTACAAGATAAGGGCTTTTTCTAGAATTCTCTACAAGCCTAGAAGATTCCAAGGACTTTCCTAGCAAATCCATAAGGATCTAGGTTTTTCCTAAGGAAATGTCCATACCTCTCTAGAATCTTCTAAAAAATGCTAGCCTTATTCTTATGTAAGCTTCCACATGGCATTAATATATGTCAAATGGCGCCTATGTGGCGGGTCATCACATCTAACTCCTAAATAATTTTAGCTCTTTAGATGAAATGTAAGCTAAGTTCTAAATAATTTAAAAGTCTTCGATATTATTTGTTAGTCTAGCAATATATACAAATTGATTGTACTAATATTCAATCAACAAGTTCAAATTAGTTGGGATACAATCCGTTATGTACTAAAATTATTCTTCTAAAAAAAGATATGTACTTATTTTAGTATCGTGCGTAGTTCTTTCAAGGTAAAAAGAGTGTATCCTAACTTAGGTACTAGAATTGATTGTACTTTTGTACGAGTTAGTTACATACATTAAACCAAACAAAGAGATTGATTGGTTACGAGATAAGTAGGAATCTGAATTGGACAGGAGAATATCCAGCGAAAGTCTCCTAATTTTTAGTTACCATACATTCAATCCAACTATATAAAGTTAAGAAAGACAGCAAAAAGTTAAAACTCCATTATTGAAAAGTTTAAAACACATTACGCATACCAAAGAAAGGCTTTGAGAGGGAGGAAGGAAACAGCTTCCTTCCAACCAAACTTCCAGTCAAAGAAATATATTCTAATATTCTTTTAA
