# Supplementary figures and images for: Foxtail Millet NF-Y Families: Genome-Wide Survey and Evolution Analyses Identified Two Functional Genes Important in Abiotic Stresses
Source: Front Plant Sci. 2015 Dec 22;6:1142. doi: 10.3389/fpls.2015.01142 (PMC4687410; doi:10.3389/fpls.2015.01142)

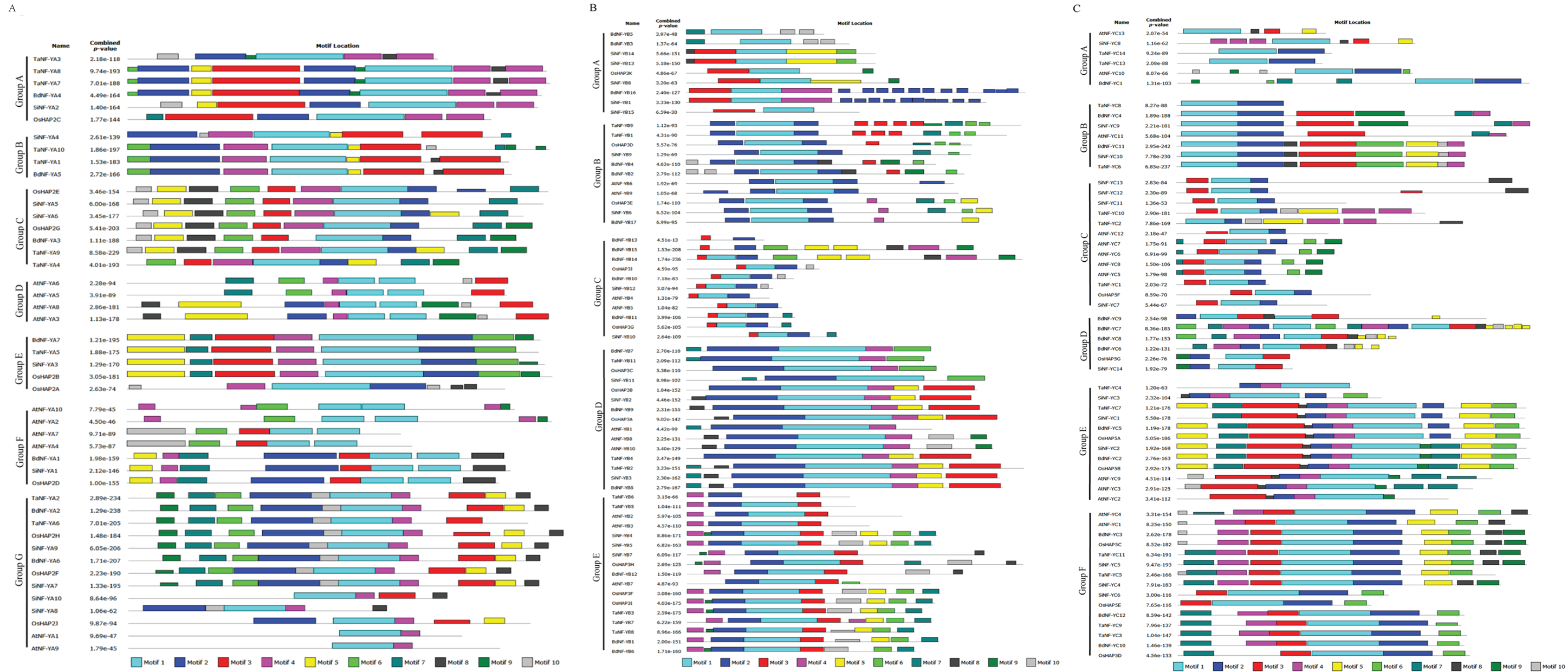

Supplement: Supplementary Figure S1 — Motifs identified for NF-Y proteins from foxtail millet, Arabidopsis, wheat, rice, and Brachypodium using MEME motif search tool. Motif structures for (A) NF-YA; (B) NF-YB; (C) NF-YC proteins. Different motifs are shown as differently colored boxes. [file Image1.TIF]

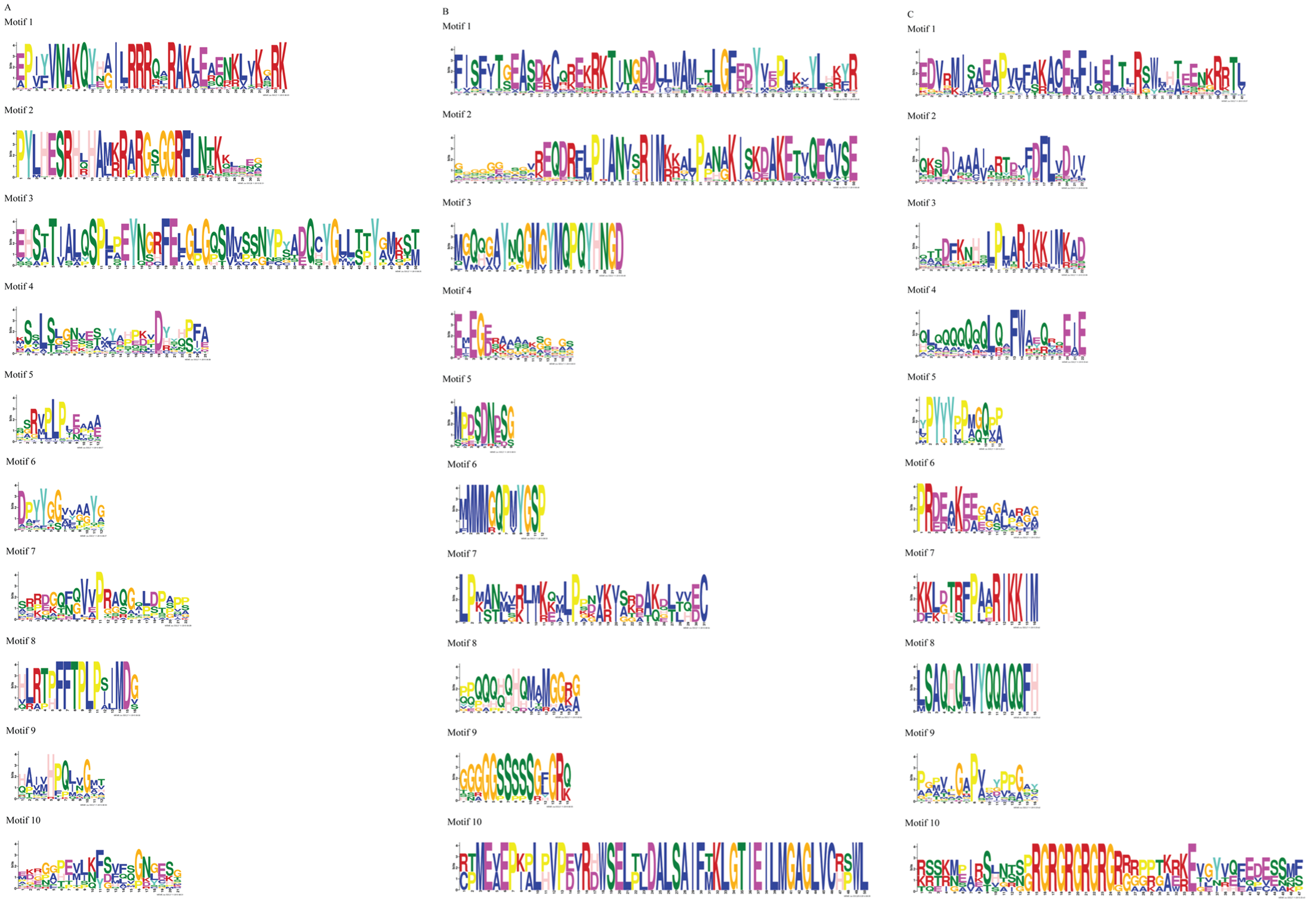

Supplement: Supplementary Figure S2 — Conserved motifs logos identified for the NF-Y proteins from foxtail millet, Arabidopsis, wheat, rice, and Brachypodium by MEME software. [file Image2.TIF]

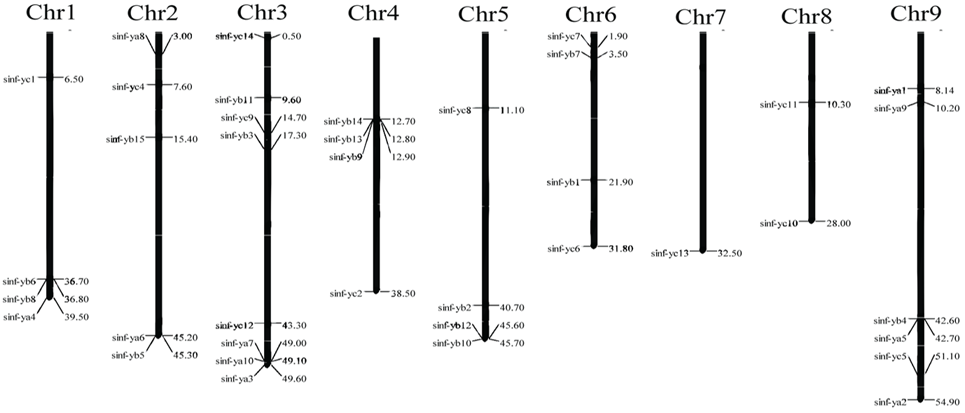

Supplement: Supplementary Figure S3 — Chromosomal locations of 39 SiNF-Y genes in foxtail millet. The chromosome numbers are indicated at the top of each bar. [file Image3.TIF]

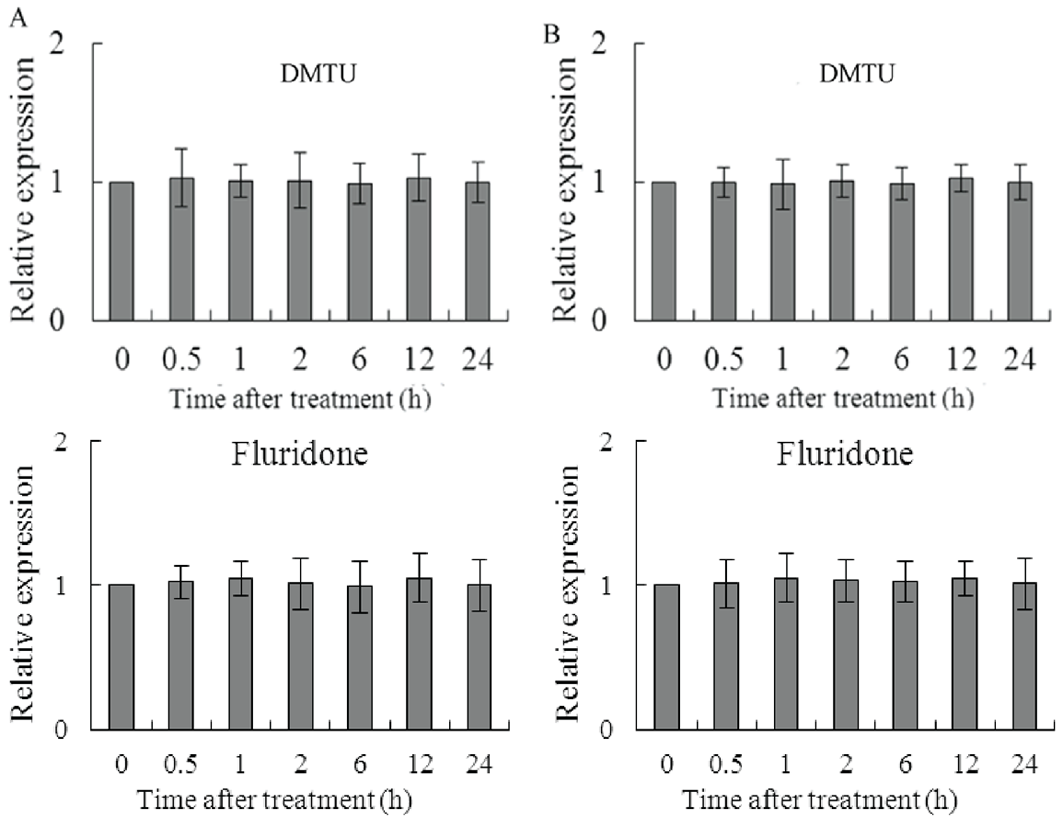

Supplement: Supplementary Figure S4 — Expression patterns of SiNF-YA1 and SiNF-YB8 under fluridone or DMTU treatment. (A) SiNF-YA1 gene; (B) SiNF-YB8 gene. Vertical bars indicate ± SE of three replicates from one sample. Three biological experiments were performed, and produced similar results. [file Image4.TIF]

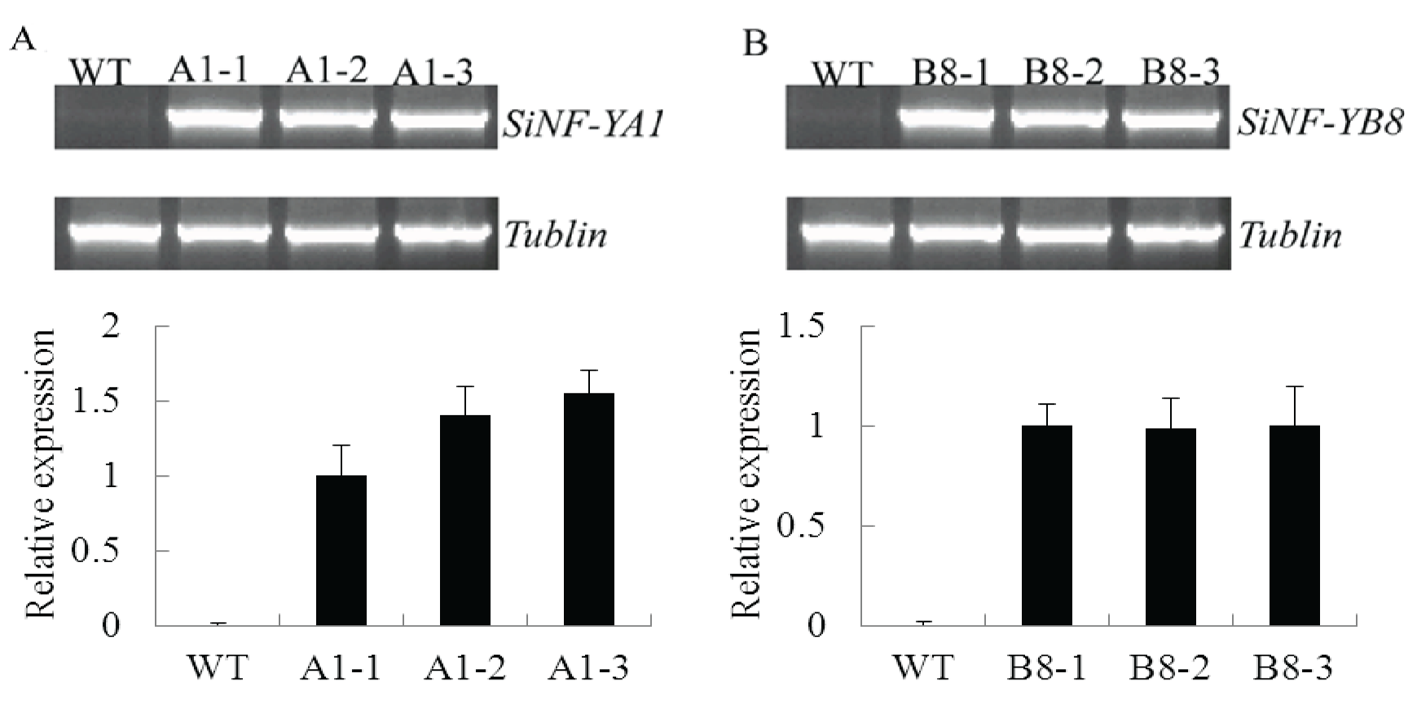

Supplement: Supplementary Figure S5 — Expression patterns of SiNF-YA1 and SiNF-YB8 in transgenic tobacco. (A) Expression of SiNF-YA1 gene detected by RT-PCR and qRT-PCR; (B) Expression of SiNF-YB8 gene detected by RT-PCR and qRT-PCR. The expression level of SiNF-YA1/SiNF-YB8 in transgenic tobacco A1-1/B8-1 was seen as 1. [file Image5.TIF]

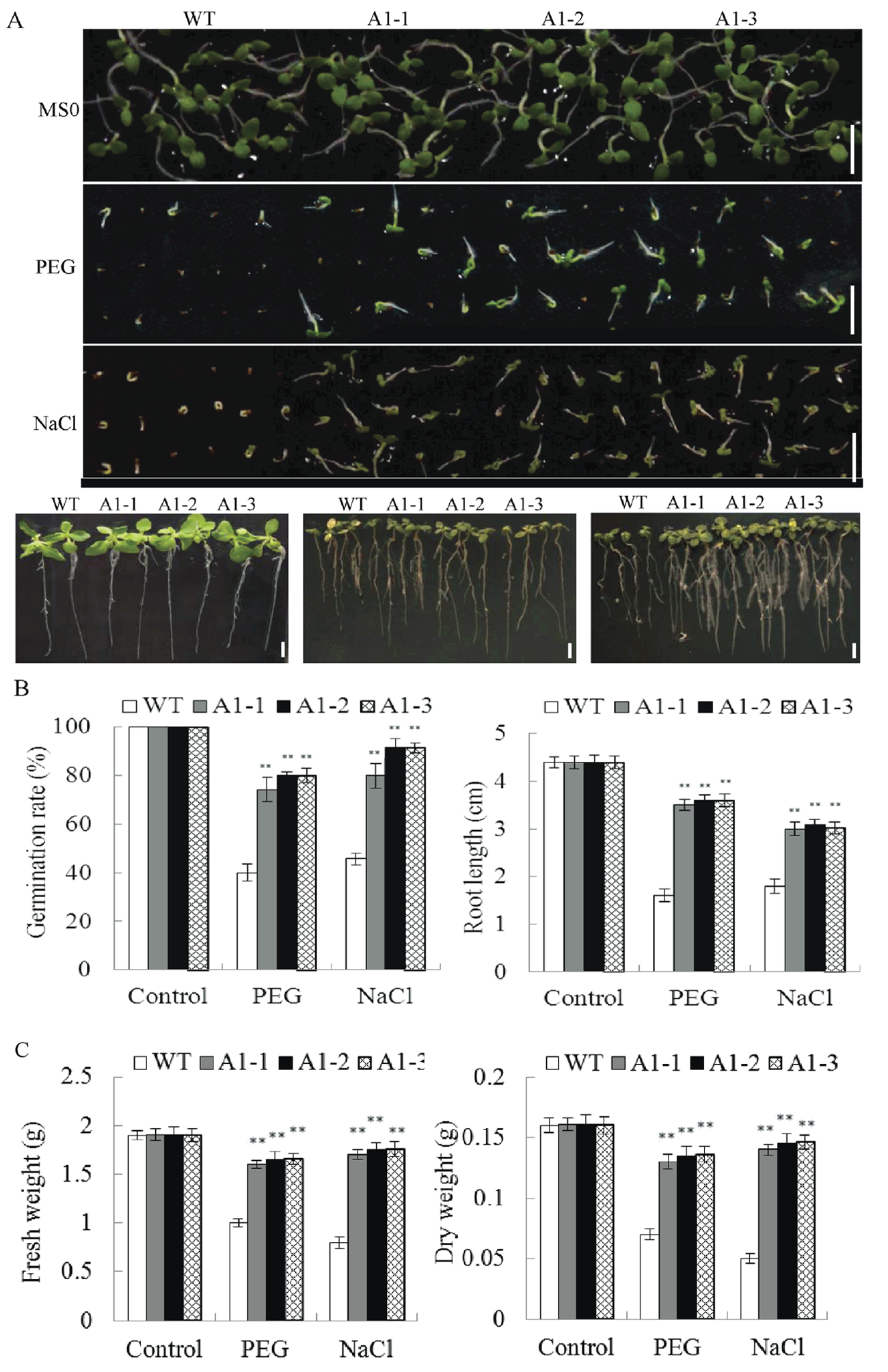

Supplement: Supplementary Figure S6 — Overexpression of SiNF-YA1 enhanced tolerance to drought and salt stresses during germination and seedling growth stages in transgenic tobacco. (A) Germination rates and root lengths of SiNF-YA1 transgenic plants grown on MS media containing PEG or NaCl. (B) Mean germination rates and root lengths. (C) Fresh weigh and dry weight. A1-1, A1-2, A1-3: 35S::SiNF-YA1 transgenic lines; WT: wild type. Vertical bars indicate ± SE of three replicates. ** indicate significant differences in comparison with the WT lines at P < 0.01. Bar, 1 cm. [file Image6.TIF]

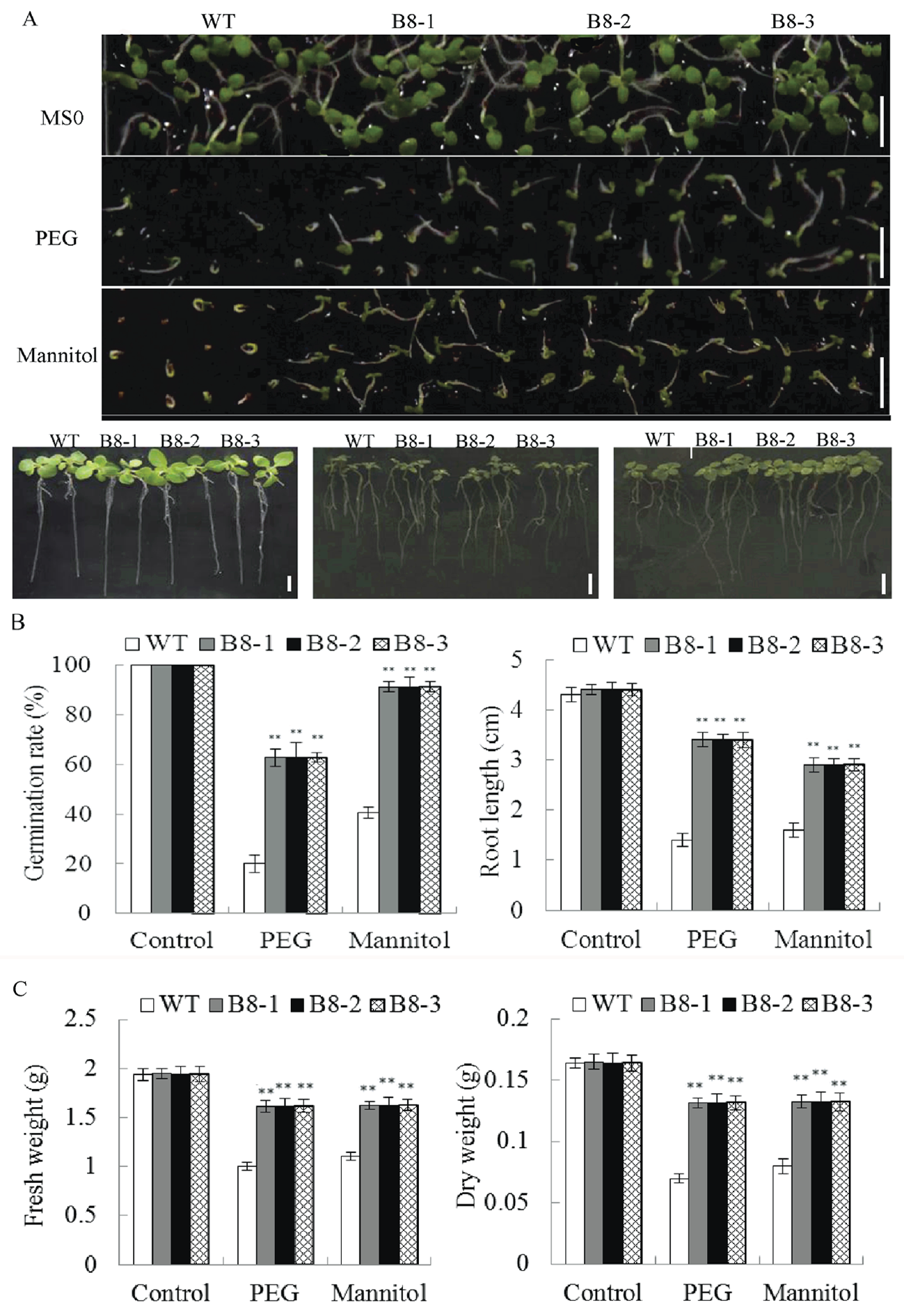

Supplement: Supplementary Figure S7 — Overexpression of SiNF-YB8 enhanced tolerance to drought and osmotic stresses during germination and seedling growth stages in transgenic tobacco. (A) Germination and root lengths of SiNF-YB8 transgenic plants were tested on MS medium containing PEG and mannitol. The seeds were allowed to grow for 6 d when the growth patterns of roots were monitored and photographed. (B) Mean germination rates and root lengths were analyzed. (C) Fresh weigh and dry weight. B8-1, B8-2, B8-3: 35S::SiNF-YB8 transgenic lines. Vertical bars indicate ± SE of three replicates. ** indicate significant differences in comparison with the WT lines at P < 0.01. Bar, 1 cm. [file Image7.TIF]
